# The immune-related microRNA miR-146b is upregulated in glioblastoma recurrence

**DOI:** 10.18632/oncotarget.25528

**Published:** 2018-06-26

**Authors:** Shariq S. Khwaja, Chunyu Cai, Shahed N. Badiyan, Xiaowei Wang, Jiayi Huang

**Affiliations:** ^1^ Department of Neurosurgery, UTHealth McGovern School of Medicine, Mischer Neuroscience Associates, Houston, TX, USA; ^2^ Department of Pathology, UT Southwestern Medical Center, Dallas, TX, USA; ^3^ Department of Radiation Oncology, University of Maryland School of Medicine, Baltimore, MD, USA; ^4^ Department of Radiation Oncology, Washington University School of Medicine, St. Louis, MO, USA

**Keywords:** miR-146b, GBM, glioma, recurrence, gene expression profiling

## Abstract

**Background:**

Glioblastoma (GBM) has a high rate of local recurrence despite chemoradiotherapy (CRT). Genome-wide expression profiling was performed on patient tumors before and after chemoradiotherapy to identify genes and gene pathways associated with recurrence.

**Results:**

Median time to recurrence was 8.9 months with median time to second surgery of 9.6 months. The microRNA (miRNA) analysis identified 9 oncologic and immune-related miRNAs to be differentially expressed, including the hypoxia-related miR-210 and the immune-modulatory miR-146b. More than 1200 differentially-expressed genes were identified with RNA-sequencing (RNA-seq). Gene set enrichment analysis (GSEA) identified p53 signaling, Notch, Wnt, VEGF, and MEK gene sets enriched in recurrent GBM. Consistent with the miRNA profiling data, the miR-146b target gene set from GSEA analysis was also associated with recurrence.

**Methods:**

Fourteen patients with GBM recurrence after CRT who had available tumor tissue from the initial diagnosis as well as recurrence were selected. Total RNA was isolated from formalin-fixed paraffin-embedded (FFPE) tumor specimens. Genome-wide expression profiling using RT-PCR for miRNA analysis and RNA-seq for messenger RNA (mRNA) analysis were conducted to identify differentially-expressed genes. GSEA was performed on the differential expression data.

**Conclusions:**

Genome-wide expression profiling identifies multiple oncologic and immune-related gene sets associated with GBM recurrence. In particular, immune-related miR-146b is upregulated in recurrence and deserves further investigation.

## INTRODUCTION

Glioblastoma (GBM, World Health Organizaiton/WHO grade IV) is the most common malignant primary brain tumor in adults and has an annual incidence of 3.2 in 100,000 in the United States [1]. Based on a large randomized study, adjuvant radiation therapy (RT) and temozolamide (TMZ) after maximal safe resection is the standard of care for GBM, but the outcomes remain dismal despite multi-modality therapy with median progression-free survival (PFS) of 6.9 months and overall survival (OS) of 14.6 months [2]. Outcomes have improved in recent clinical trials with median OS of approximately 17 months [3, 4]. Tumor recurrence occurs predominantly inside the radiation field rather than distantly [5], suggesting a subpopulation of GBM cells that are highly resistant to chemoradiotherapy (CRT). With recurrence, treatment is more limited and outcomes remain poor [6]. Recent advances in immunotherapy have generated excitement in developing novel approaches to treat this devastating disease [7, 8]. For example, programmed-death-1 (PD-1), an immune checkpoint surface receptor expressed on lymphocytes, has been implicated as a mediator of immune suppression by tumors such as GBM [8]. Nivolumab, an anti-PD-1 checkpoint inhibitor, is currently under investigation in two randomized studies for newly diagnosed GBM (NCT02617589 and NCT02667587). However, chemoradiotherapy for GBM can result in severe and prolonged lymphopenia that may contribute to tumor recurrence and interfere with future success of immunotherapy [9, 10]. Thus, there is a clear need to better understand the mechanisms of GBM resistance to CRT and its interaction with immune system, which will be crucial for the discovery of novel therapies for this devastating disease. However, due to the difficulty of access to GBM tissue, particularly recurrent tumor tissue, detailed molecular profiling to compare changes in tumors before and after CRT have been limited.

MicroRNAs (miRNAs) are a family of small non-coding RNA molecules (∼22 nucleotides) that regulate expression of thousands of protein-coding genes [11]. With the ability to regulate the expression of a large number of genes, miRNAs function as master regulators of important processes such as cell growth, apoptosis, cancer initiation and progression [12, 13]. Functional studies with modulation of certain miRNAs have demonstrated direct effects on glioma stem cell properties and sensitivity to CRT [14–17]. There is also increasing evidence that miRNAs play important regulatory roles on the immune pathways currently targeted by the major immunotherapeutic approaches, including immune checkpoint blockade, adoptive cell transfer, cancer vaccines, and cytokine therapy [18].

Given the importance of miRNAs, we hypothesized that miRNA expression changes in GBM after CRT may allow an improved understanding of pathways responsible for treatment resistance. We also employed a genome-wide expression profiling approach of 14 GBM tumors before CRT and their corresponding recurrent tumor after CRT to identify oncologic and immune-related changes within the recurrent tumors after CRT.

## RESULTS

### Patients and outcomes

Fourteen patients were eligible for the analysis (Supplementary Table 1). There were 7 females and 7 males included in this study. The mean age was 53 years. Patients received RT with concurrent temozolomide followed by adjuvant temozolomide from 2006-2012. The median dose to the planning target volume was 60 Gy. Two patients had placement of carmustine implants (Gliadel wafer; Eisai Inc) at the time of initial surgery and one patient received concurrent bevacizumab. After recurrence but before the second salvage surgery, three patients received salvage bevacizumab-based chemotherapy, and one patient received laser interstitial thermal therapy (LITT). Median time to progression was 8.9 months (range: 2.9–24.4). Median time to second surgery was 10.3 months (3.4–32.9). Only one patient was alive at the time of this analysis. Median OS was 23.1 months (12.3–72.0). O^6^-methylguanine-DNA-methyltransferase (MGMT) methylation and isocitrate dehydrogenase (IDH) status were largely not assessed in this patient cohort.

### miRNA profiling identifies oncologic and immune-related miRNAs

miRNA profiling was performed for the initial and recurrent tumors for all 14 patients. A real-time reverse transcriptase-polymerase chain reaction (RT-PCR) based miRNA profiling experiment revealed 9 miRNAs differentially expressed with statistical significance in recurrent GBM (Table 1). The miRNA with the greatest fold change was miR-210 (recurrence/initial ratio 1.91, *p =* 0.003), which has established roles in cell survival, particularly in hypoxic conditions [23]. miR-146b, which has been shown to be a critical mediator of immune-regulatory pathways [24, 25], was also upregulated (recurrence/initial ratio 1.55, *p =* 0.02). Table 1 also shows several select predicted targets of the miRNAs based on miRDB prediction (http://mirdb.org) [26], which correspond to oncologic and immune-related genes, including *Notch2* [27], *TGFBR1* [28], *STAT3* [29], *FGFRL1* [30], and *NOVA1* [31].

**Table 1:** miRNA differentially expressed in recurrent high-grade glioma

miRNA	Fold change	*p*-value	Select predicted targets (mirdb)
hsa-mir-210	1.91	0.003	IGF2, FGFRL1, AIFM3, CEND1
hsa-mir-191	1.44	0.005	TMOD2 (neuronal), MAPK9, CRCAM, CDK6, Notch2, DAPK1, TRAF3, BDNF
hsa-mir-101	1.66	0.005	TGFBR1, GLTSCR1
hsa-mir-126^*^	0.44	0.016	MACC1, MAP3K2, TNFAIP8L3, RASAL2, CCNT2, MAPK10, TRAF6
hsa-mir-96	0.33	0.018	MTSS1, SDC2, NEUROD4, MAP2K1, RAB35
hsa-mir-146b	1.55	0.024	ITPR2, RGS4, IL1A, NAV3, NOVA1, SRSF6
hsa-mir-25	0.72	0.024	CD69, JMY, RAB23, MYO1B, MAP2K4
hsa-mir-24	1.41	0.031	CALCR, AAK1, KSR2
hsa-mir-20a	0.69	0.034	MAP3K2, PDCD1LG2, AAK1, TNFRSF21, NEDD4L

Fold change represents the mean expression value of recurrent glioma divided by initial tumor. *P*-value calculated using a paired Student’s *T*-test.

### RNA-seq analysis

Given the multiple oncologic and immune-related miRNAs differentially expressed in recurrent GBM, a large-scale genome-wide expression profiling study using RNA-sequencing (RNA-seq) was employed to identify genes and gene pathways associated with recurrence. Due to limited RNA quality from two patients as well as the initial tumor of a third patient, RNA-seq was employed on 11 initial and 12 recurrent RNA samples. More than 1200 genes were identified that were differentially expressed with statistical significance (*p <* 0.05) (Supplementary Table 2). Selected genes related to iron-metabolism, oncologic pathways, and immune function are shown in Table 2, including transferrin (*TF*), ferritin (*FTL*), fibroblast growth factor 1 (*FGF1*), TNF receptor-associated factor 4 (*TRAF4*), and signal transducer and activator of transcription 1 (*STAT1*).

**Table 2:** Select differentially-expressed genes in recurrent GBM

*Upregulated in recurrence*			
Gene name	Gene symbol	Relative expression	*P*-value
transferrin	TF	5.34	0.0085
interferon-induced protein with tetratricopeptide repeats 2	IFIT2	3.54	0.0006
apoptosis-associated tyrosine kinase	AATK	3.38	0.0125
cyclin-dependent kinase 18	CDK18	3.30	0.0292
breast carcinoma amplified sequence 1	BCAS1	2.94	0.0221
microtubule-associated protein 7	MAP7	2.59	0.0101
fibroblast growth factor receptor 2	FGFR2	2.42	0.0400
ferritin, light polypeptide	FTL	2.35	0.0025
fibroblast growth factor 1	FGF1	2.22	0.0058
signal transducer and activator of transcription 1	STAT1	2.08	0.0019
lysosomal-associated membrane protein 2	LAMP2	1.78	0.0008

*Downregulated in recurrence*			
Gene name	Gene symbol	Relative expression	*P*-value
neurocan	NCAN	0.26	0.0433
glutamate receptor, ionotropic, AMPA 2	GRIA2	0.45	0.0334
fatty acid binding protein 7, brain	FABP7	0.48	0.0236
ets variant 1	ETV1	0.49	0.0209
nestin	NES	0.52	0.0370
TNF receptor-associated factor 4	TRAF4	0.53	0.0334
melanoma antigen family D, 4	MAGED4	0.54	0.0076
neuro-oncological ventral antigen 1	NOVA1	0.55	0.0330

*P*-values calculated using a two-sided Student’s *T*-Test

### GSEA implicates multiple oncologic and immune-related genes in recurrence

**Figure 1:**
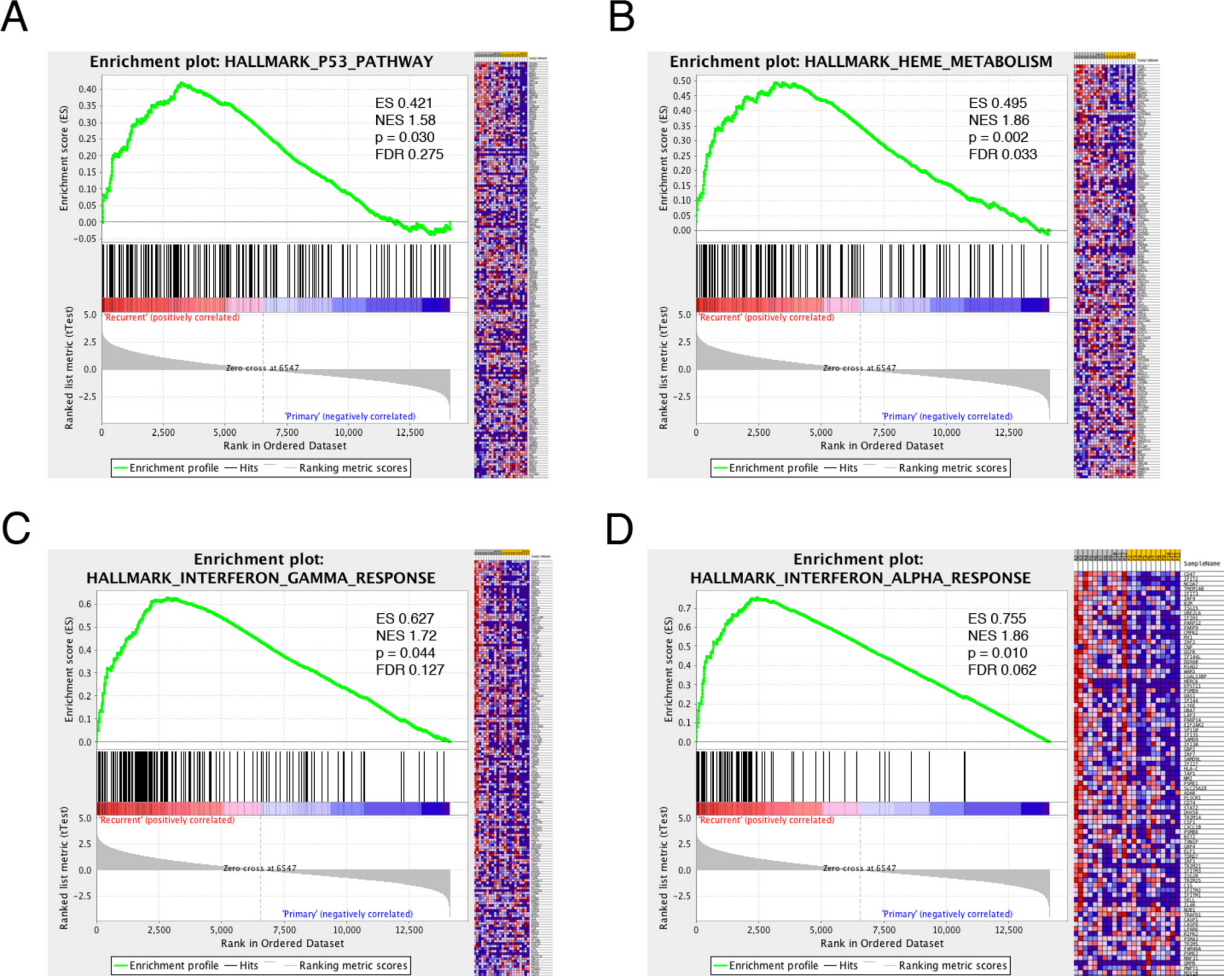
Gene Set Enrichment Analysis (GSEA) in recurrent glioma GSEA of differential expression between recurrent and initial high-grade glioma demonstrates that recurrent GBM is enriched in genes that participate in the p53 tumor suppressor pathway (**A**), heme metabolism (**B**) and interferon signaling (**C–D**). ES–enrichment score, NES–normalized enrichment score, FDR–false discovery ratio.

**Figure 2:**
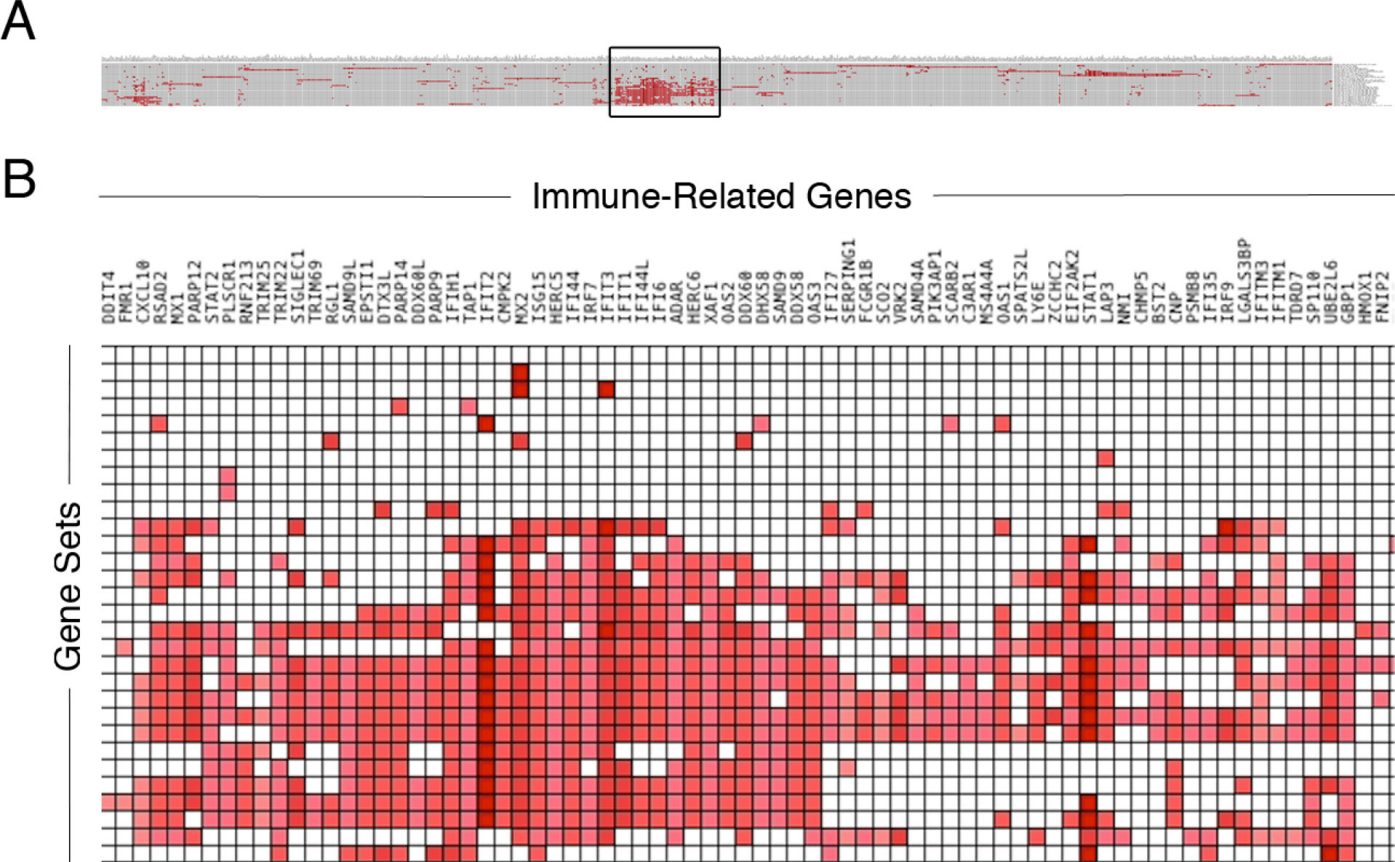
Immune-related genes associated with recurrent glioma Genes differentially expressed in recurrent gliomas enriched across multiple immunologic gene sets. Investigation of the top most enriched genes in the various immunologic gene sets from the GSEA (**A**) results in the boxed overlap region (**B**).

Given the significant number of genes differentially expressed, gene set enrichment analysis (GSEA) was employed to identify pathways associated with recurrence. Using this statistical tool, recurrent GBM was noted to be enriched in gene sets associated with the MEK, KRAS, Notch, VEGF, and Wnt pathways, among others (Supplementary Table 3). Additionally, the gene sets associated with P53, iron metabolism, and interferon signaling were associated with GBM recurrence (Figure 1A–1D). Given the association between GBM recurrence and immune-related genes and gene sets, we explored the immunologic story further. Multiple immune-related gene sets were associated with recurrence (Figure 1C–1D and data not shown). Using the Leading Edge analysis in the GSEA statistical software package [22] (Figure 2A), an overlap region corresponding to multiple immune-related genes present in multiple enriched gene sets was identified (Figure 2B). Multiple immune-regulatory interferon-related genes as well as STAT1/2 (Signal Transducer and Activator of Transcription) in particular, which plays a key role in tumor progression [32–34] and immune-modulation [29, 35–37], were present in this overlap region.

### miR-146b gene set identified in GBM recurrence

**Figure 3:**
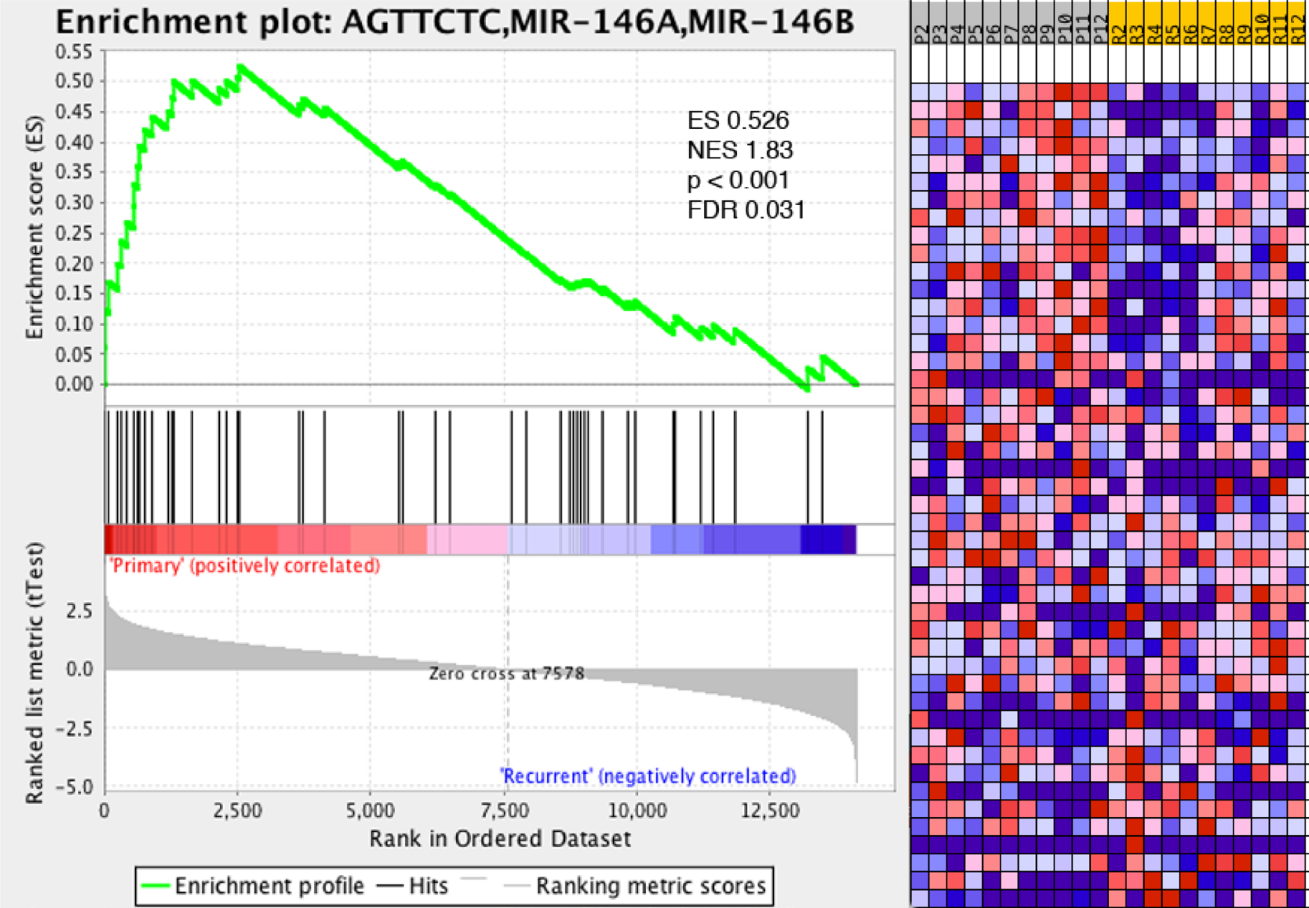
Immune-related gene set miR-146b associated with recurrent glioma GSEA reveals gene set associated with targets of miR-146b associated with recurrent glioma, linking miRNA profiling results with RNA-seq gene expression analysis. ES–enrichment score, NES–normalized enrichment score, FDR–false discovery ratio.

We previously demonstrated increased expression levels of the immune-modulatory miRNA, miR-146b, in tumor tissue isolated from recurrent GBM (Table 1). Interestingly, GSEA analysis of all miRNA gene sets revealed that the gene set corresponding to miR-146b targets was found to be enriched in GBM recurrence (Figure 3). Multiple immune-related targets of miR-146b have been identified by miRDB prediction (http://mirdb.org). Supplementary Table 4 shows several miR-146b target immune-related genes with decreased expression from the RNA-seq dataset comparing gene expression between recurrent and initial tumors, including Nova1, HNRNPD/AUF1A, and SNX22.

### miR-146b fold change associated with local recurrence

**Figure 4:**
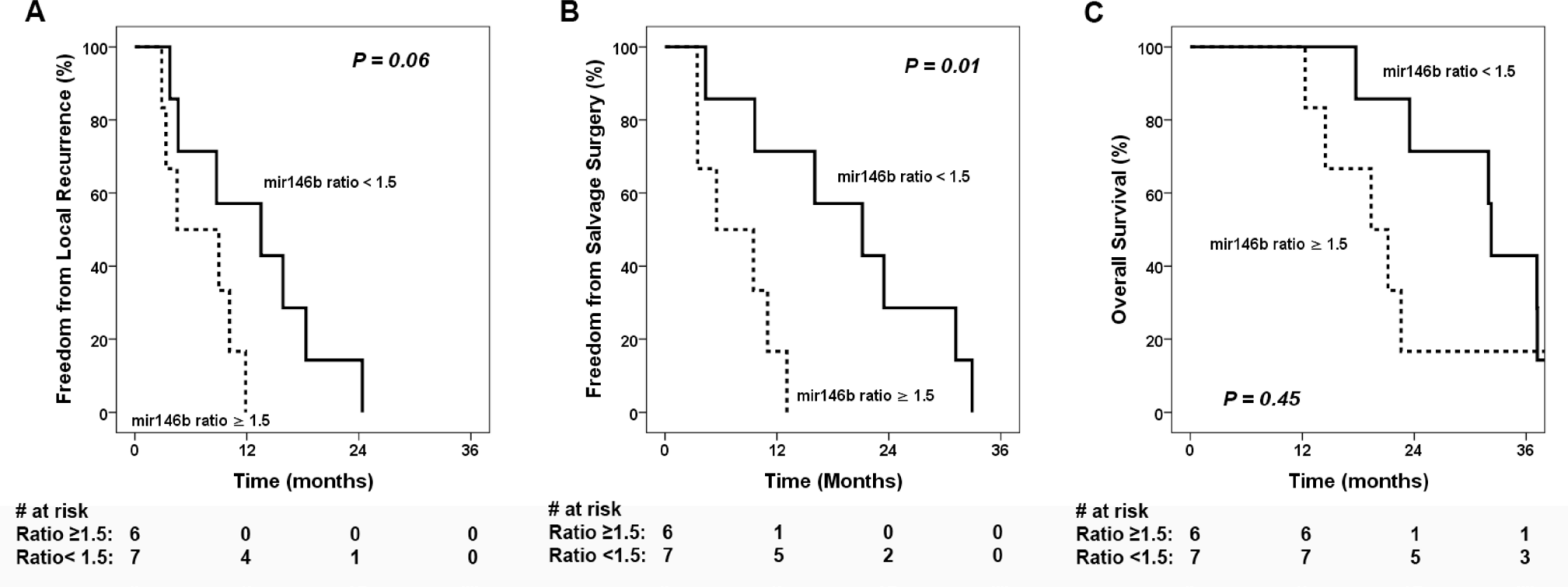
Clinical outcomes stratified by miR-146b expression ratio Kaplan-Meier estimates of clinical outcomes stratified by high and low miR-146b ratios between recurrent vs initial tumors (**A**) freedom from local recurrence, (**B**) freedom from salvage surgery, and (**C**) overall survival.

The median fold change of miR-146b between recurrent and initial tumors was 1.5 (range: 0.6–5.4). Low miR-146b change (ratio < 1.5) was associated with significantly improved freedom from salvage surgery ( FFSS) (Figure 4B, *p =* 0.01) and a trend toward improved freedom from local recurrence (FFLR) (Figure 4A, *p =* 0.06). However, the local control did not translate to significant difference on OS (*p =* 0.45, Figure 4C). Cox regression analysis (Supplementary Table 5) using miR-146b ratio as a continuous variable showed an association with time to local recurrence (hazard ratio [HR] 1.59, 95% confidence interval [CI] 1.007–2.52, *p =* 0.047) as well as time to salvage surgery (HR 1.68, 95% CI 1.06–2.69, *p =* 0.03), but not with time to death (HR 1.32, 95% CI 0.86–2.04, *p =* 0.21).

## DISCUSSION

This pilot study compared miRNA and mRNA expression profiles of 14 pairs of GBM tumors before CRT and after recurrence. It identified several candidate oncologic and immune-related miRNAs and gene sets differentially expressed in recurrent GBM. In particular, the immune-modulatory miRNA miR-146b was significantly increased at recurrence and may be a promising target for future studies. Due to the difficulty after repeated biopsy of intracranial tumors, detailed expression profiling studies of miRNA and transcriptome (or RNA) of GBM tumors before and after CRT have been limited in the literature. Park *et al.* previously performed a miRNA analysis on 2 untreated GBM tumors and 2 recurrent GBM tumors after CRT (from two different patients) where they identified 43 miRNAs with at least two-fold changes in expression levels in both of the recurrent tumors (miR-146b is not among them) [38]. However, given the recurrent and initial tumors were not from the same patients and the extremely limited sample size, it’s difficult to interpret this study’s findings. Bo *et al.* [39] analyzed the publically available miRNA profile data from 12 paired primary and recurrent GBM samples using microarrays. They noted that miR-146b was downregulated in the recurrent samples. However, the treatments of these 12 patients before their recurrences were not described, so there may be inherent differences between the patient populations. Furthermore, the discrepancy may be due to the differences in the miRNA profiling techniques. The PCR-based platform in the current study has shown greater detection specificity (at single-base level) than the microarray platform, and it is commonly used to validate microarray or RNA-seq results. Other studies have conducted genome and exome sequencing of larger cohorts of paired initial and recurrent GBM tumors, but they did not specifically analyze miRNA expression changes [40, 41]. These studies paint complex pictures of mutation alterations and evolutionary trajectories of GBM tumors during CRT but do not explore the potential of common miRNA pathways in GBM resistance. The differentially higher expression of miR-146b in recurrent GBM found in our analysis of paired recurrent and initial GBM is in contradiction to investigations from Li *et al.* [42], Katakowski *et al.* [43], Liu *et al.* [44] and Yang *et al.* [45], who all demonstrated that overexpression of miR-146b in a GBM cell culture system reduces migration/invasion and increases apoptosis and radiosensitivity. These results need to be taken with the necessary caveats of an *in vitro* culture system which inherently lacks the complexity of GBM recurrence *in vivo*. Our hypothesis presented in this manuscript is that miR-146b upregulation in recurrence plays a role in GBM immune-suppression potentially at a later stage of the tumor progression event, ultimately resulting in tumor recurrence. This manuscript challenges the notion of miR-146b functioning as solely a tumor suppressor. Based on our investigation of human GBM recurrent tumor tissue in relation to the paired initial tumor, the role of miR-146b in tumorigenesis, tumor progression, and tumor maintenance is likely more complex than has been previously reported in the literature.

A vast number of potential targetable pathways in GBM have been identified in preclinical studies, but few have shown efficacy in the clinical setting. Complicating clinical efficacy is the heterogeneity of these tumors stemming from the complexity of their molecular makeup [41, 46]. This complexity is highlighted in the results presented in this study using a Gene Set Enrichment Analysis (GSEA) with recurrent GBM enriched in MEK, NOTCH, SRC, RAF, P53, VEGF, and WNT gene sets. Studies have shown redundant activation of numerous signaling pathways, which likely explains the futility of single-drug therapies [47].

This study implicates the immune-modulatory miRNA miR-146b as an important factor to drive GBM recurrence after chemoradiotherapy. Multiple studies have established an immune-modulatory role for miR-146b, including suppression of IL-6 allowing p16-dependent tumorigenesis in breast cancer [24], as well as suppression of multiple other pro-inflammatory cytokines and chemokines such as TNF-alpha, IL-8, and IL-10 [25]. Park *et al.* implicated miR-146b in dendritic cell survival and cytokine production via targeting of TRAF6 and IRAK1 [48]. Lu *et al.* also recently showed that miRNA-146b are highly expressed in regulatory T cells (Tregs) and play a vital role in their expansion, survival, and suppressor function [49]. Since Tregs have been implicated as a major contributor to the evasion of immunosurveillance by GBM and are correlated with poor prognosis [50], our finding may be a reflection of the miRNA changes of the infiltrating immune cells within the tumor microenvironment rather than the tumor cells. Our gene expression results highlight several established miR-146b immune-modulatory targets, including HNRNPD/AUF1A and SNX22 (Supplementary Table 4). HNRNPD/AUF1A is a nuclear ribonucleoprotein with roles in NF-kB signaling and interleukin expression [51]. SNX22 has been shown through its role in endocytosis to down-regulate CD4 [52]. As immunotherapy continues to develop for GBM, an improved understanding of the role of miR-146b may help to develop novel approaches to overcome the high level of immune suppression by the GBM microenvironment [53–55].

Since this study is a small pilot study of genome-expression profiling, its findings need to be interpreted with the following limitations. As this study is based on a limited sample size and a highly selected population of GBM patients who underwent salvage surgery for recurrence after CRT, the finding should be considered hypothesis-generating. The small sample size limits the use of stringent FDR-controlling method. External validation using a larger sample size, ideally using a multi-institutional tumor bank, will be necessary to confirm our finding. Recent data have also shown significant regional and cellular heterogeneity within GBM [56, 57]. Therefore, the expression profiling of our samples may only represent snap-shots of the complex composition of the tumor cells and tumor-infiltrating immune cells, and other methods such as single-cell RNA-seq to compare tumor cells before and after treatment will be needed to provide validation in the future. Furthermore, our study does not prove causality that the expression change of miR-146b represents the mechanism driving the recurrence rather than passive treatment-induced changes. Functional studies *in vitro* and *in vivo* will be needed to further confirm the role of miR-146b in promoting GBM recurrence.

In conclusion, this study presents multiple deregulated miRNAs and gene pathways after CRT and implicates the immune-modulatory miR-146b as a potential factor in GBM recurrence. Additional investigations are warranted to validate the association of miR-146b with GBM recurrence and to understand its mechanisms.

## MATERIALS AND METHODS

### Patient selection

Eligible patients with pathologically diagnosed GBM were identified from our institutional review board (IRB)–approved database. They were required to have received standard fractionated RT and concurrent daily TMZ at 75 mg/m^2^ after maximal safe resection. These patients were also required to have sufficient pathologic tissue from the initial surgery before CRT and the salvage surgery at the time of recurrence after CRT. Additional salvage chemotherapies were allowed prior to the salvage surgery. This study was reviewed and granted approval by the IRB.

### RNA isolation from tumor specimens

Formalin-fixed paraffin-embedded (FFPE) tumor blocks from the initial and salvage surgeries were collected for pathological analysis. FFPE specimens were stained with hematoxylin and eosin (H&E), reviewed by a board-certified neuro-pathologist (CC) to confirm diagnosis and to demarcate tumor regions. This was followed by macrodissection of 10-micron unstained sections in order to remove non-tumor tissues. Total RNA was then extracted using the miRNeasy FFPE Kit (Qiagen) according to the manufacturer’s protocol.

### miRNA profiling

miRNA expression profiling of 96 miRNAs implicated in tumorigenesis was performed using our laboratory’s previously described method, which is based on RT-PCR, the details of which have been described previously [19]. The RT reaction was performed with the High Capacity cDNA Reverse Transcription Kit (Applied Biosystems, Foster City, CA). Each RT reaction included 150 ng of tumor RNA and miRNA-specific RT primers. RT-PCR was performed with Power SYBR Green PCR Master Mix (Applied Biosystems) and miRNA-specific PCR primers. Raw profiling data based on PCR threshold cycles (Ct) were normalized using a quantile-based scaling method, which has been described previously [19].

### RNA-seq for gene expression profiling in recurrent and initial GBM

RNA-seq was performed to allow identification and quantification of expression of genes in a given tumor sample by a single massively parallel sequencing run [20]. Details of the experimental protocol has been described previously [21]. Total RNA was used to construct multiplexed cDNA libraries, which were provided to the Genome Technology Access Center (GTAC) at Washington University School of Medicine for sequencing. Ribosomal RNA (rRNA) was removed using the RiboMinus kit (Life Technologies) and custom designed rRNA probes. rRNA-depleted total RNA was used as template for RNA-seq library construction using the NEBNext mRNA Library Prep kit (New England BioLabs). Double-stranded cDNA was synthesized from rRNA-depleted total RNA, end-repaired, dA-tailed, and then ligated to standard Illumina adaptor oligonucleotides. Adaptor-ligated cDNA libraries were loaded into HiSeq 2000 (Illumina) for sequencing. Raw RNA-seq data (in fastq format) were made available after the sequencing runs, which were parsed to align each sequence read to the original RNA sample. Sequencing reads were modified to remove low quality reads and clustered before mapping to the human transcriptomes. Gene expression levels were normalized using a strategy based on the length of the transcript as well as the number of total read counts from each sample (reads per kilobase per million, RPKM).

### Gene Set Enrichment Analysis (GSEA)

Differential gene expression profiles were generated using RNA-seq from recurrent GBM (*n =* 12) and the initial tumor (*n =* 11). Utilizing GSEA software available from the Broad Institute [22], gene sets with member genes enriched in recurrent GBM were discovered. Statistically significant gene sets were considered with *p*-value < 0.05 and FDR (false discovery ratio) < 0.25. ES (enrichment score) reflects the degree to which a gene set is over-represented in our dataset of differentially expressed genes. NES (normalized enrichment score) is the ES normalized for gene set size.

### Statistical analysis

All time points were determined from the date of initial surgery. Statistical significance (*p <* 0.05) for differential expression was calculated using the Student’s *T*-test. Median value of the fold change in miR-146b expression in recurrent vs initial tumors was used as cutoff to identify high fold change (mir-146b ratio ≥ 1.5) vs low fold change (mir-146b ratio < 1.5). Freedom from local recurrence (FFLR), freedom from salvage surgery (FFSS), and overall survival (OS) were evaluated using the Kaplan-Meier method and compared using the log-rank test. Cox regression analysis was utilized to evaluate the association of miR-146b fold change with clinical outcomes. Statistical analyses were performed with the Statistical Package for Social Sciences, version 22 (IBM SPSS Statistics, Chicago, IL, USA). Significance was defined as a *p* value ≤ 0.05. All statistical tests were two sided.

## SUPPLEMENTARY MATERIALS TABLES






