# Supplementary material for: The immune-related microRNA miR-146b is upregulated in glioblastoma recurrence
Source: Oncotarget. 2018 Jun 26;9(49):29036–46. doi: 10.18632/oncotarget.25528 (PMC6044384; doi:10.18632/oncotarget.25528)
Supplement: Supplementary file 1 [file oncotarget-09-29036-s001.pdf]

The immune-related microRNA miR-146b is upregulated in glioblastoma recurrence

SUPPLEMENTARY MATERIALS

Supplementary Table 1: Patient, tumor, and treatment characteristics. See Supplementary_Table_1

Supplementary Table 2: All statistically significant differentially-expressed genes in recurrent GBM. See Supplementary_Table_2

Supplementary Table 3: GSEA oncologic gene sets enriched in recurrent GBM

Gene set	Description	ES	NES	*P*-value	FDR
MEK	Genes down-regulated by MEK upregulation	0.42	1.83	<0.001	0.187
KRAS	Genes down-regulated by Ras upregulation	0.49	1.64	0.026	0.242
NOTCH	Genes up-regulated by Notch down-regulation	0.43	1.62	0.021	0.180
SRC	Genes up-regulated by Src down-regulation	0.41	1.61	0.019	0.179
ALK	Genes down-regulation by ALK down-regulation	0.46	1.61	0.016	0.169
RAF	Genes up-regulated by Raf up-regulation	0.45	1.61	0.021	0.157
P53	Genes down-regulated by P53 down-regulation	0.44	1.59	0.020	0.148
AKT	Genes up-regulated by AKT up-regulation	0.42	1.57	0.026	0.156
VEGF	Genes up-regulated by VEGF up-regulation	0.41	1.51	0.061	0.196
WNT	Genes down-regulated by Wnt up-regulation	0.37	1.50	0.040	0.200
CYCLIN D1	Genes down-regulated by Cyclin D1 down-regulation	0.36	1.50	0.031	0.195
PTEN	Genes down-regulated by PTEN down-regulation	0.37	1.51	0.026	0.206

Abbreviations: ES - enrichment score, NES - normalized enrichment score, FDR - false discovery ratio, GSEA - gene set enrichment analysis.

Supplementary Table 4: Select miR-146b target gene differential expression from RNA-seq analysis

miR-146b target	Ratio (R/P)	*p*-value	Known function
SRSF6	0.72	0.008	mRNA splicing, negative regulation of cell death
NOVA1	0.55	0.033	neuron-specific RNA-binding protein, tumor suppressor in astrocytoma
KCTD15	0.60	0.002	neural crest development
HNRNPD/AUF1A	0.69	0.003	nuclear ribonucleoprotein, mRNA stability, NF-kB pathway, IL expression
SNX22	0.75	0.047	intracellular trafficking, CD4 cell surface expression
NS1-BP	0.63	0.026	c-Myc transcriptional control

Ratio R/P refers normalized gene expression value in recurrence divided by normalized gene expression value in the initial tumor. *P*-value calculated using a Student's *T*-test.

Supplementary Table 5: Cox regression analysis of association of miR-146b with clinical outcomes

	HR (95% CI)	*p*-value
Time to local recurrence	1.592 (1.007–2.516)	0.047
Time to salvage therapy	1.684 (1.056–2.686)	0.028
Time to death	1.321 (0.856–2.037)	0.208

HR - hazard ratio, CI - confidence interval.
