# Supplementary material for: The immune-related microRNA miR-146b is upregulated in glioblastoma recurrence
Source: Oncotarget. 2018 Jun 26;9(49):29036–46. doi: 10.18632/oncotarget.25528 (PMC6044384; doi:10.18632/oncotarget.25528)
Supplement: Supplementary file 2 [file oncotarget-09-29036-s002.docx]

| **Supplementary Table 1: Patient, Tumor, and Treatment Characteristics** | | | | | | |  |  |  |  |  |  |  |  |
| --- | --- | --- | --- | --- | --- | --- | --- | --- | --- | --- | --- | --- | --- | --- |
|  |  |  |  | |  |  |  |  |  |  |  |  |  |  |
| **Patient** | **Gender** | **Age** | **Histology** | | **MGMT** | **IDH** | **RT Dose** | **Concurrent Chemo** | **Adjuvant Therapy** | **Time to Progression (mo)** | **Time to 2nd surgery (mo)** | **Salvage Therapy before 2nd surgery** | **OS (mo)** | **Alive at last follow-up** |
|  |  |  |  | |  |  |  |  |  |  |  |  |  |  |
| 1 | Female | 57 | GBM | | not assessed | not assessed | 6000 | temozolamide | temozolamide | 15.9 | 16.1 | none | 23.5 | no |
| 2 | Male | 43 | GBM | | not assessed | not assessed | 6000 | temozolamide | temozolamide | 2.9 | 3.5 | none | 19.4 | no |
| 3 | Male | 71 | GBM | | not assessed | not assessed | 6000 | temozolamide | temozolamide | 9.0 | 9.5 | none | 12.3 | no |
| 4 | Female | 43 | GBM | | not assessed | not assessed | 6000 | temozolamide | temozolamide | 4.5 | 5.5 | none | 22.6 | no |
| 5 | Female | 57 | GBM | | not assessed | not assessed | 6000 | temozolamide | temozolamide | 8.7 | 9.6 | none | 50.9 | no |
| 6 | Female | 54 | GBM | | not assessed | not assessed | 6300 | temozolamide | temozolamide | 3.7 | 4.3 | none | 17.7 | no |
| 7 | Female | 51 | GBM | | unmethylated | not assessed | 6000 | temozolamide + bevacizumab | temozolamide + bevacizumab | 11.9 | 13.1 | none | 21.2 | no |
| 8 | Female | 62 | GBM | | methylated | not mutated | 6000 | temozolamide | temozolamide | 24.4 | 32.9 | laser interstitial thermal therapy | 37.2 | no |
| 9 | Male | 55 | GBM | | unmethylated | mutated | 6000 | temozolamide | temozolamide | 10.2 | 11.0 | none | 14.5 | no |
| 10 | Male | 26 | GBM | | unmethylated | not assessed | 6000 | temozolamide + carmustine | temozolamide + carmustine | 3.3 | 3.4 | none | 72.0 | yes |
| 11 | Male | 46 | GBM | | unmethylated | not mutated | 6300 | temozolamide | temozolamide | 18.3 | 31.2 | bevacizumab-based chemo | 37.2 | no |
| 12 | Female | 57 | GBM | | unmethylated | not assessed | 6300 | temozolamide | temozolamide | 5.9 | 6.6 | none | 17.5 | no |
| 13 | Male | 64 | GBM | | methylated | not assessed | 6000 | temozolamide | temozolamide | 4.6 | 21.2 | bevacizumab-based chemo | 32.3 | no |
| 14 | Male | 56 | GBM | | unmethylated | not mutated | 6300 | temozolamide | temozolamide | 13.5 | 23.5 | bevacizumab-based chemo | 32.0 | no |
|  |  |  |  | |  |  |  |  |  |  |  |  |  |  |
| Abbreviations: MGMT - O6-Methylguanine-DNA methyltransferase, IDH - isocitrate dehydrogenase, RT - radiation therapy, OS - overall survival | | | | | | | | | |  |  |  |  |  |
| Age represents age at time of first surgery | | | |  |  |  |  |  |  |  |  |  |  |  |
| Time to progression, time to second surgery, and overall survival calculated from the date of the initial surgery | | | | | | | | |  |  |  |  |  |  |
