# Supplementary material for: The immune-related microRNA miR-146b is upregulated in glioblastoma recurrence
Source: Oncotarget. 2018 Jun 26;9(49):29036–46. doi: 10.18632/oncotarget.25528 (PMC6044384; doi:10.18632/oncotarget.25528)
Supplement: Supplementary file 3 [file oncotarget-09-29036-s003.docx]

| **Supplementary Table 2: All statistically significant differentially-expressed genes in recurrent GBM** | | | | | |
| --- | --- | --- | --- | --- | --- |
|  |  |  |  |  |  |
|  |  |  |  |  |  |
| **T-Test** | **Ratio** | **Primary** | **Recur** | **Gene** | **Gene Detail** |
|  |  |  |  |  |  |
| 0.00010 | 1.408 | 5.5 | 7.8 | FAM45A | family with sequence similarity 45, member A |
| 0.00016 | 0.670 | 15.0 | 10.0 | MEX3C | mex-3 RNA binding family member C |
| 0.00017 | 2.106 | 8.4 | 17.6 | OPTN | optineurin |
| 0.00029 | 1.650 | 18.0 | 29.7 | CD47 | CD47 molecule |
| 0.00035 | 0.669 | 14.1 | 9.5 | ZNF587B | zinc finger protein 587B |
| 0.00039 | 1.507 | 10.8 | 16.3 | CCDC53 | coiled-coil domain containing 53 |
| 0.00049 | 0.650 | 22.4 | 14.6 | F2R | coagulation factor II (thrombin) receptor |
| 0.00050 | 1.336 | 5.3 | 7.1 | TMEM254 | transmembrane protein 254 |
| 0.00059 | 0.720 | 25.3 | 18.2 | ZNF587 | zinc finger protein 587 |
| 0.00060 | 0.664 | 13.9 | 9.2 | AC092295.7 | uncharacterized LOC101927599 |
| 0.00061 | 3.537 | 6.2 | 21.8 | IFIT2 | interferon-induced protein with tetratricopeptide repeats 2 |
| 0.00064 | 1.635 | 10.4 | 17.1 | NPC1 | Niemann-Pick disease, type C1 |
| 0.00065 | 0.677 | 14.3 | 9.7 | STK36 | serine/threonine kinase 36 |
| 0.00085 | 1.785 | 33.7 | 60.2 | LAMP2 | lysosomal-associated membrane protein 2 |
| 0.00087 | 1.642 | 6.2 | 10.3 | TPRG1L | tumor protein p63 regulated 1-like |
| 0.00089 | 0.611 | 10.9 | 6.7 | ZNF283 | zinc finger protein 283 |
| 0.00092 | 0.778 | 19.1 | 14.9 | PCF11 | PCF11 cleavage and polyadenylation factor subunit |
| 0.00095 | 0.773 | 10.3 | 8.0 | NCKAP5L | NCK-associated protein 5-like |
| 0.00096 | 3.135 | 23.7 | 74.4 | RNASE1 | ribonuclease, RNase A family, 1 (pancreatic) |
| 0.00112 | 1.635 | 21.2 | 34.7 | MAPRE2 | microtubule-associated protein, RP/EB family, member 2 |
| 0.00114 | 1.661 | 12.6 | 20.9 | NR1D2 | nuclear receptor subfamily 1, group D, member 2 |
| 0.00117 | 0.673 | 27.8 | 18.7 | SPDYE1 | speedy/RINGO cell cycle regulator family member E1 |
| 0.00121 | 0.592 | 24.7 | 14.6 | ZNF154 | zinc finger protein 154 |
| 0.00125 | 0.749 | 15.5 | 11.6 | SETDB1 | SET domain, bifurcated 1 |
| 0.00127 | 0.750 | 29.6 | 22.2 | FUS | fused in sarcoma |
| 0.00131 | 0.682 | 50.1 | 34.2 | LUC7L | LUC7-like (S. cerevisiae) |
| 0.00134 | 0.752 | 20.8 | 15.7 | ASXL1 | additional sex combs like 1 (Drosophila) |
| 0.00134 | 0.716 | 20.7 | 14.8 | ZNF506 | zinc finger protein 506 |
| 0.00137 | 1.463 | 26.6 | 38.8 | SERINC3 | serine incorporator 3 |
| 0.00139 | 2.426 | 6.4 | 15.5 | FAM134B | family with sequence similarity 134, member B |
| 0.00139 | 0.833 | 37.1 | 30.9 | ANKRD17 | ankyrin repeat domain 17 |
| 0.00141 | 0.633 | 19.5 | 12.3 | DZIP1 | DAZ interacting zinc finger protein 1 |
| 0.00153 | 1.413 | 14.6 | 20.7 | SFXN3 | sideroflexin 3 |
| 0.00154 | 0.632 | 17.0 | 10.7 | ZNF552 | zinc finger protein 552 |
| 0.00154 | 1.551 | 10.9 | 16.9 | STX7 | syntaxin 7 |
| 0.00157 | 1.354 | 5.7 | 7.7 | SSTR5-AS1 | SSTR5 antisense RNA 1 |
| 0.00165 | 2.069 | 7.8 | 16.0 | SYNGR2 | synaptogyrin 2 |
| 0.00171 | 1.227 | 11.6 | 14.3 | USP32 | ubiquitin specific peptidase 32 |
| 0.00173 | 1.915 | 8.2 | 15.8 | SYTL2 | synaptotagmin-like 2 |
| 0.00177 | 1.610 | 11.1 | 17.8 | TEX2 | testis expressed 2 |
| 0.00181 | 0.656 | 10.9 | 7.2 | ZNF75A | zinc finger protein 75a |
| 0.00183 | 0.800 | 17.9 | 14.3 | DIDO1 | death inducer-obliterator 1 |
| 0.00186 | 0.769 | 9.5 | 7.3 | NOP2 | NOP2 nucleolar protein |
| 0.00190 | 3.837 | 37.1 | 142.2 | PTGDS | prostaglandin D2 synthase 21kDa (brain) |
| 0.00192 | 1.828 | 7.0 | 12.7 | GCA | grancalcin, EF-hand calcium binding protein |
| 0.00193 | 2.085 | 31.1 | 64.8 | STAT1 | signal transducer and activator of transcription 1, 91kDa |
| 0.00197 | 0.755 | 30.4 | 23.0 | TPR | translocated promoter region, nuclear basket protein |
| 0.00199 | 0.771 | 27.2 | 21.0 | ARID1A | AT rich interactive domain 1A (SWI-like) |
| 0.00206 | 1.207 | 12.8 | 15.4 | FNIP1 | folliculin interacting protein 1 |
| 0.00213 | 1.547 | 11.1 | 17.2 | DUSP3 | dual specificity phosphatase 3 |
| 0.00219 | 0.767 | 23.7 | 18.1 | B3GALNT2 | beta-1,3-N-acetylgalactosaminyltransferase 2 |
| 0.00221 | 1.517 | 22.4 | 34.0 | NCOA4 | nuclear receptor coactivator 4 |
| 0.00222 | 0.701 | 8.3 | 5.8 | ZNF616 | zinc finger protein 616 |
| 0.00223 | 1.402 | 7.1 | 9.9 | RPL23AP7 | ribosomal protein L23a pseudogene 7 |
| 0.00227 | 1.735 | 8.4 | 14.5 | GAS6 | growth arrest-specific 6 |
| 0.00234 | 0.750 | 9.8 | 7.4 | CRAMP1L | Crm, cramped-like (Drosophila) |
| 0.00244 | 0.687 | 22.0 | 15.1 | DPY19L3 | dpy-19-like 3 (C. elegans) |
| 0.00248 | 2.311 | 450.2 | 1040.6 | FTL | ferritin, light polypeptide |
| 0.00249 | 0.774 | 11.3 | 8.7 | NFATC2IP | nuclear factor of activated T-cells, cytoplasmic, calcineurin-dependent 2 interacting protein |
| 0.00254 | 1.440 | 5.2 | 7.5 | NR1D1 | nuclear receptor subfamily 1, group D, member 1 |
| 0.00255 | 0.599 | 11.2 | 6.7 | KCTD15 | potassium channel tetramerization domain containing 15 |
| 0.00263 | 1.494 | 18.8 | 28.0 | TECR | trans-2,3-enoyl-CoA reductase |
| 0.00267 | 1.637 | 11.0 | 18.0 | VAMP1 | vesicle-associated membrane protein 1 (synaptobrevin 1) |
| 0.00270 | 1.483 | 13.8 | 20.4 | GHITM | growth hormone inducible transmembrane protein |
| 0.00270 | 1.833 | 17.3 | 31.7 | LGMN | legumain |
| 0.00276 | 0.698 | 8.8 | 6.1 | OSGEP | O-sialoglycoprotein endopeptidase |
| 0.00279 | 0.719 | 85.5 | 61.5 | CCNL2 | cyclin L2 |
| 0.00285 | 0.716 | 20.6 | 14.7 | PIP5K1A | phosphatidylinositol-4-phosphate 5-kinase, type I, alpha |
| 0.00288 | 0.484 | 18.7 | 9.0 | LOC101927308 | uncharacterized LOC101927308 |
| 0.00288 | 2.032 | 24.8 | 50.4 | SLC44A1 | solute carrier family 44 (choline transporter), member 1 |
| 0.00290 | 0.680 | 21.8 | 14.8 | ZFP30 | ZFP30 zinc finger protein |
| 0.00295 | 1.483 | 24.5 | 36.3 | ATP6V1E1 | ATPase, H+ transporting, lysosomal 31kDa, V1 subunit E1 |
| 0.00295 | 1.600 | 8.2 | 13.1 | ARL2 | ADP-ribosylation factor-like 2 |
| 0.00296 | 1.439 | 10.3 | 14.8 | TMEM140 | transmembrane protein 140 |
| 0.00297 | 2.179 | 9.0 | 19.5 | RARRES3 | retinoic acid receptor responder (tazarotene induced) 3 |
| 0.00312 | 1.362 | 7.9 | 10.7 | STRN | striatin, calmodulin binding protein |
| 0.00312 | 0.747 | 7.5 | 5.6 | ZNF28 | zinc finger protein 28 |
| 0.00314 | 1.313 | 5.1 | 6.7 | LOC148696 | uncharacterized LOC148696 |
| 0.00314 | 0.680 | 29.2 | 19.9 | PLEKHG2 | pleckstrin homology domain containing, family G (with RhoGef domain) member 2 |
| 0.00314 | 1.644 | 6.3 | 10.3 | SNX30 | sorting nexin family member 30 |
| 0.00319 | 1.946 | 22.4 | 43.7 | CD9 | CD9 molecule |
| 0.00325 | 2.003 | 17.3 | 34.7 | CREG1 | cellular repressor of E1A-stimulated genes 1 |
| 0.00334 | 1.957 | 6.9 | 13.5 | NCOA7 | nuclear receptor coactivator 7 |
| 0.00335 | 1.229 | 17.4 | 21.3 | UBE2Z | ubiquitin-conjugating enzyme E2Z |
| 0.00336 | 1.518 | 5.3 | 8.0 | FUCA1 | fucosidase, alpha-L- 1, tissue |
| 0.00337 | 1.443 | 6.1 | 8.8 | C11orf71 | chromosome 11 open reading frame 71 |
| 0.00343 | 0.685 | 57.0 | 39.0 | HNRNPD | heterogeneous nuclear ribonucleoprotein D (AU-rich element RNA binding protein 1, 37kDa) |
| 0.00345 | 0.779 | 29.5 | 23.0 | RSRC2 | arginine/serine-rich coiled-coil 2 |
| 0.00353 | 0.713 | 63.3 | 45.1 | COA1 | cytochrome c oxidase assembly factor 1 homolog (S. cerevisiae) |
| 0.00353 | 1.487 | 22.4 | 33.3 | EPS15 | epidermal growth factor receptor pathway substrate 15 |
| 0.00354 | 1.228 | 11.0 | 13.5 | DMXL1 | Dmx-like 1 |
| 0.00355 | 0.769 | 12.8 | 9.8 | TTC28 | tetratricopeptide repeat domain 28 |
| 0.00356 | 2.311 | 56.2 | 129.9 | CTSD | cathepsin D |
| 0.00361 | 1.294 | 12.3 | 16.0 | CRBN | cereblon |
| 0.00369 | 0.729 | 16.8 | 12.2 | NFE2L3 | nuclear factor, erythroid 2-like 3 |
| 0.00370 | 1.293 | 13.2 | 17.0 | C6orf106 | chromosome 6 open reading frame 106 |
| 0.00376 | 0.685 | 10.3 | 7.1 | ZNF776 | zinc finger protein 776 |
| 0.00377 | 0.737 | 292.6 | 215.6 | HNRNPA2B1 | heterogeneous nuclear ribonucleoprotein A2/B1 |
| 0.00399 | 2.166 | 19.4 | 42.0 | IFIT3 | interferon-induced protein with tetratricopeptide repeats 3 |
| 0.00403 | 1.300 | 17.5 | 22.7 | KIAA0368 | KIAA0368 |
| 0.00404 | 1.362 | 5.2 | 7.1 | ZCCHC2 | zinc finger, CCHC domain containing 2 |
| 0.00406 | 1.608 | 11.2 | 18.0 | ANXA7 | annexin A7 |
| 0.00409 | 0.708 | 37.4 | 26.5 | SPDYE5 | speedy/RINGO cell cycle regulator family member E5 |
| 0.00410 | 1.551 | 15.0 | 23.2 | IRF9 | interferon regulatory factor 9 |
| 0.00412 | 0.691 | 13.0 | 9.0 | ZNF544 | zinc finger protein 544 |
| 0.00413 | 0.748 | 23.3 | 17.5 | CSNK1E | casein kinase 1, epsilon |
| 0.00414 | 0.661 | 21.7 | 14.3 | ZNF814 | zinc finger protein 814 |
| 0.00424 | 1.669 | 28.4 | 47.3 | ENO2 | enolase 2 (gamma, neuronal) |
| 0.00425 | 1.472 | 7.5 | 11.1 | GYG1 | glycogenin 1 |
| 0.00435 | 1.936 | 12.9 | 25.1 | CTSL | cathepsin L |
| 0.00441 | 0.713 | 8.6 | 6.2 | RCOR1 | REST corepressor 1 |
| 0.00443 | 1.506 | 6.5 | 9.9 | PER3 | period circadian clock 3 |
| 0.00447 | 0.712 | 13.9 | 9.9 | ADCY6 | adenylate cyclase 6 |
| 0.00450 | 1.708 | 8.0 | 13.6 | CRYL1 | crystallin, lambda 1 |
| 0.00466 | 0.539 | 14.4 | 7.7 | ANKRD50 | ankyrin repeat domain 50 |
| 0.00466 | 1.777 | 8.2 | 14.5 | C2 | complement component 2 |
| 0.00468 | 1.435 | 7.2 | 10.4 | ZER1 | zyg-11 related, cell cycle regulator |
| 0.00469 | 1.588 | 6.2 | 9.9 | VRK2 | vaccinia related kinase 2 |
| 0.00479 | 1.367 | 11.1 | 15.2 | ZCRB1 | zinc finger CCHC-type and RNA binding motif 1 |
| 0.00482 | 0.649 | 24.9 | 16.1 | PRMT1 | protein arginine methyltransferase 1 |
| 0.00484 | 1.419 | 10.0 | 14.2 | C18orf32 | chromosome 18 open reading frame 32 |
| 0.00495 | 0.757 | 15.9 | 12.0 | DROSHA | drosha, ribonuclease type III |
| 0.00499 | 1.462 | 7.2 | 10.6 | UROS | uroporphyrinogen III synthase |
| 0.00507 | 1.802 | 6.9 | 12.4 | PHLDA3 | pleckstrin homology-like domain, family A, member 3 |
| 0.00509 | 1.300 | 31.0 | 40.3 | MTCH1 | mitochondrial carrier 1 |
| 0.00513 | 2.152 | 9.1 | 19.6 | LPAR1 | lysophosphatidic acid receptor 1 |
| 0.00518 | 2.145 | 9.3 | 19.8 | FAM107B | family with sequence similarity 107, member B |
| 0.00524 | 0.673 | 10.6 | 7.1 | ZNF219 | zinc finger protein 219 |
| 0.00527 | 0.760 | 27.2 | 20.7 | PSPC1 | paraspeckle component 1 |
| 0.00530 | 0.738 | 15.6 | 11.5 | MRPS14 | mitochondrial ribosomal protein S14 |
| 0.00531 | 1.390 | 5.3 | 7.4 | GABPB2 | GA binding protein transcription factor, beta subunit 2 |
| 0.00532 | 1.840 | 148.4 | 273.1 | PSAP | prosaposin |
| 0.00532 | 1.745 | 7.0 | 12.2 | TCP11L2 | t-complex 11, testis-specific-like 2 |
| 0.00533 | 0.829 | 11.0 | 9.1 | EIF4ENIF1 | eukaryotic translation initiation factor 4E nuclear import factor 1 |
| 0.00534 | 0.610 | 14.9 | 9.1 | DOT1L | DOT1-like histone H3K79 methyltransferase |
| 0.00538 | 0.646 | 16.6 | 10.7 | GDF11 | growth differentiation factor 11 |
| 0.00538 | 0.669 | 35.4 | 23.7 | NASP | nuclear autoantigenic sperm protein (histone-binding) |
| 0.00544 | 1.739 | 8.3 | 14.5 | ALAD | aminolevulinate dehydratase |
| 0.00546 | 0.689 | 11.7 | 8.1 | LMBR1L | limb development membrane protein 1-like |
| 0.00552 | 0.767 | 32.1 | 24.6 | UPF3A | UPF3 regulator of nonsense transcripts homolog A (yeast) |
| 0.00553 | 1.625 | 6.4 | 10.5 | EGLN3 | egl-9 family hypoxia-inducible factor 3 |
| 0.00556 | 1.770 | 33.4 | 59.2 | PMP22 | peripheral myelin protein 22 |
| 0.00562 | 0.632 | 33.6 | 21.2 | CCDC14 | coiled-coil domain containing 14 |
| 0.00564 | 1.569 | 21.0 | 32.9 | UBE2L6 | ubiquitin-conjugating enzyme E2L 6 |
| 0.00565 | 1.680 | 7.4 | 12.5 | FAM53B | family with sequence similarity 53, member B |
| 0.00569 | 1.277 | 10.3 | 13.1 | ATG3 | autophagy related 3 |
| 0.00572 | 0.734 | 9.9 | 7.3 | TIMM44 | translocase of inner mitochondrial membrane 44 homolog (yeast) |
| 0.00573 | 0.758 | 51.2 | 38.8 | HNRNPDL | heterogeneous nuclear ribonucleoprotein D-like |
| 0.00578 | 0.611 | 9.9 | 6.1 | LOC101930310 | uncharacterized LOC101930310 |
| 0.00580 | 1.284 | 13.3 | 17.1 | KLHL24 | kelch-like family member 24 |
| 0.00580 | 2.223 | 15.7 | 34.9 | FGF1 | fibroblast growth factor 1 (acidic) |
| 0.00581 | 0.784 | 14.4 | 11.3 | HDAC8 | histone deacetylase 8 |
| 0.00589 | 1.305 | 5.9 | 7.7 | RAB11FIP2 | RAB11 family interacting protein 2 (class I) |
| 0.00591 | 1.823 | 29.4 | 53.7 | MAP1A | microtubule-associated protein 1A |
| 0.00591 | 1.615 | 6.4 | 10.3 | TLE4 | transducin-like enhancer of split 4 |
| 0.00592 | 0.696 | 24.1 | 16.8 | TET2 | tet methylcytosine dioxygenase 2 |
| 0.00595 | 0.798 | 10.7 | 8.5 | ZC3H4 | zinc finger CCCH-type containing 4 |
| 0.00601 | 0.624 | 9.5 | 5.9 | SLC6A1-AS1 | SLC6A1 antisense RNA 1 |
| 0.00611 | 2.487 | 22.6 | 56.1 | SEPP1 | selenoprotein P, plasma, 1 |
| 0.00618 | 0.735 | 11.3 | 8.3 | CSNK1E | LOC400927-CSNK1E readthrough |
| 0.00628 | 0.783 | 16.9 | 13.2 | DEDD | death effector domain containing |
| 0.00629 | 0.712 | 11.8 | 8.4 | LOC102724559 | uncharacterized LOC102724559 |
| 0.00631 | 0.717 | 11.9 | 8.5 | YPEL1 | yippee-like 1 (Drosophila) |
| 0.00635 | 0.710 | 11.8 | 8.4 | MASP2 | mannan-binding lectin serine peptidase 2 |
| 0.00640 | 1.827 | 10.7 | 19.5 | NINJ1 | ninjurin 1 |
| 0.00646 | 1.253 | 51.8 | 64.9 | RAB7A | RAB7A, member RAS oncogene family |
| 0.00652 | 1.327 | 5.0 | 6.7 | ANXA4 | annexin A4 |
| 0.00662 | 1.807 | 8.8 | 15.9 | GSTO1 | glutathione S-transferase omega 1 |
| 0.00663 | 0.741 | 16.1 | 11.9 | AMOTL1 | angiomotin like 1 |
| 0.00664 | 0.743 | 8.2 | 6.1 | PTP4A3 | protein tyrosine phosphatase type IVA, member 3 |
| 0.00676 | 1.246 | 17.6 | 22.0 | SEC62 | SEC62 homolog (S. cerevisiae) |
| 0.00681 | 1.644 | 11.7 | 19.3 | MEGF9 | multiple EGF-like-domains 9 |
| 0.00681 | 1.866 | 16.5 | 30.7 | LANCL1 | LanC lantibiotic synthetase component C-like 1 (bacterial) |
| 0.00692 | 1.591 | 11.7 | 18.6 | BAIAP2 | BAI1-associated protein 2 |
| 0.00693 | 2.219 | 9.9 | 22.0 | FAIM2 | Fas apoptotic inhibitory molecule 2 |
| 0.00693 | 2.446 | 9.0 | 22.0 | TYMP | thymidine phosphorylase |
| 0.00697 | 0.636 | 14.9 | 9.5 | ZNF692 | zinc finger protein 692 |
| 0.00698 | 1.617 | 5.7 | 9.2 | ENPP4 | ectonucleotide pyrophosphatase/phosphodiesterase 4 (putative) |
| 0.00699 | 0.754 | 65.5 | 49.4 | SUGP2 | SURP and G patch domain containing 2 |
| 0.00714 | 0.685 | 9.5 | 6.5 | ZNF551 | zinc finger protein 551 |
| 0.00714 | 1.984 | 27.0 | 53.5 | CD68 | CD68 molecule |
| 0.00716 | 1.962 | 573.6 | 1125.6 | B2M | beta-2-microglobulin |
| 0.00717 | 0.607 | 39.1 | 23.7 | LOC102724363 | uncharacterized LOC102724363 |
| 0.00719 | 0.652 | 13.4 | 8.7 | ZNF211 | zinc finger protein 211 |
| 0.00728 | 2.092 | 9.4 | 19.6 | SEMA4D | sema domain, immunoglobulin domain (Ig), transmembrane domain (TM) and short cytoplasmic domain, (semaphorin) 4D |
| 0.00729 | 1.682 | 5.7 | 9.5 | PLA2G4C | phospholipase A2, group IVC (cytosolic, calcium-independent) |
| 0.00733 | 2.375 | 11.5 | 27.3 | CADM3 | cell adhesion molecule 3 |
| 0.00749 | 0.773 | 11.6 | 9.0 | ZDHHC8 | zinc finger, DHHC-type containing 8 |
| 0.00753 | 0.791 | 6.5 | 5.2 | RP11-118E18.4 | uncharacterized LOC101927539 |
| 0.00756 | 1.807 | 10.2 | 18.3 | CBR1 | carbonyl reductase 1 |
| 0.00760 | 1.344 | 10.2 | 13.7 | PLEKHM1 | pleckstrin homology domain containing, family M (with RUN domain) member 1 |
| 0.00760 | 0.541 | 18.6 | 10.1 | MAGED4 | melanoma antigen family D, 4 |
| 0.00762 | 1.670 | 10.8 | 18.0 | PFKP | phosphofructokinase, platelet |
| 0.00776 | 1.391 | 5.0 | 7.0 | AMN1 | antagonist of mitotic exit network 1 homolog (S. cerevisiae) |
| 0.00779 | 0.731 | 8.6 | 6.3 | ZNF100 | zinc finger protein 100 |
| 0.00784 | 1.528 | 11.4 | 17.4 | CD99L2 | CD99 molecule-like 2 |
| 0.00793 | 1.646 | 378.1 | 622.4 | FTH1 | ferritin, heavy polypeptide 1 |
| 0.00796 | 1.525 | 5.5 | 8.4 | YPEL2 | yippee-like 2 (Drosophila) |
| 0.00801 | 0.487 | 14.2 | 6.9 | LOC102724182 | uncharacterized LOC102724182 |
| 0.00804 | 1.901 | 5.0 | 9.6 | FOLR2 | folate receptor 2 (fetal) |
| 0.00807 | 3.221 | 8.1 | 26.1 | EDIL3 | EGF-like repeats and discoidin I-like domains 3 |
| 0.00810 | 1.497 | 12.3 | 18.5 | RNH1 | ribonuclease/angiogenin inhibitor 1 |
| 0.00811 | 1.605 | 5.6 | 9.0 | CCR1 | chemokine (C-C motif) receptor 1 |
| 0.00813 | 1.415 | 7.0 | 9.9 | TTC7B | tetratricopeptide repeat domain 7B |
| 0.00813 | 2.656 | 5.5 | 14.5 | S100A1 | S100 calcium binding protein A1 |
| 0.00814 | 0.687 | 12.9 | 8.9 | ZNF195 | zinc finger protein 195 |
| 0.00814 | 1.370 | 13.8 | 18.9 | NAGK | N-acetylglucosamine kinase |
| 0.00824 | 1.432 | 31.0 | 44.3 | WBP2 | WW domain binding protein 2 |
| 0.00826 | 1.447 | 9.2 | 13.3 | GALC | galactosylceramidase |
| 0.00830 | 0.617 | 15.5 | 9.6 | PABPC1L | poly(A) binding protein, cytoplasmic 1-like |
| 0.00838 | 2.114 | 10.9 | 23.1 | CCDC152 | coiled-coil domain containing 152 |
| 0.00838 | 4.221 | 6.6 | 28.0 | ENPP2 | ectonucleotide pyrophosphatase/phosphodiesterase 2 |
| 0.00841 | 0.713 | 56.7 | 40.4 | SRSF6 | serine/arginine-rich splicing factor 6 |
| 0.00845 | 0.777 | 28.5 | 22.2 | ZBED6 | zinc finger, BED-type containing 6 |
| 0.00845 | 1.339 | 5.0 | 6.7 | SH3D21 | SH3 domain containing 21 |
| 0.00847 | 5.340 | 11.1 | 59.3 | TF | transferrin |
| 0.00851 | 0.548 | 22.1 | 12.1 | SNORA11E | small nucleolar RNA, H/ACA box 11E |
| 0.00858 | 1.719 | 31.2 | 53.7 | SQSTM1 | sequestosome 1 |
| 0.00861 | 0.740 | 14.2 | 10.5 | ZNF260 | zinc finger protein 260 |
| 0.00862 | 1.897 | 8.2 | 15.6 | MFAP3L | microfibrillar-associated protein 3-like |
| 0.00863 | 1.759 | 18.4 | 32.5 | RNF13 | ring finger protein 13 |
| 0.00864 | 0.748 | 10.7 | 8.0 | BMS1 | BMS1 ribosome biogenesis factor |
| 0.00866 | 2.227 | 41.3 | 92.1 | SCD | stearoyl-CoA desaturase (delta-9-desaturase) |
| 0.00874 | 0.643 | 18.0 | 11.6 | CHST11 | carbohydrate (chondroitin 4) sulfotransferase 11 |
| 0.00875 | 1.286 | 9.4 | 12.1 | XPC | xeroderma pigmentosum, complementation group C |
| 0.00881 | 1.545 | 8.2 | 12.7 | IGSF8 | immunoglobulin superfamily, member 8 |
| 0.00882 | 0.757 | 14.8 | 11.2 | HKR1 | HKR1, GLI-Kruppel zinc finger family member |
| 0.00888 | 0.694 | 14.4 | 10.0 | ZNF586 | zinc finger protein 586 |
| 0.00891 | 0.803 | 14.1 | 11.3 | ZNF333 | zinc finger protein 333 |
| 0.00897 | 0.680 | 13.7 | 9.3 | CIRH1A | cirrhosis, autosomal recessive 1A (cirhin) |
| 0.00902 | 0.607 | 10.6 | 6.4 | ZNF675 | zinc finger protein 675 |
| 0.00904 | 1.475 | 24.2 | 35.8 | ARL6IP5 | ADP-ribosylation-like factor 6 interacting protein 5 |
| 0.00915 | 0.762 | 7.5 | 5.7 | MIER3 | mesoderm induction early response 1, family member 3 |
| 0.00916 | 4.218 | 7.6 | 32.1 | TPPP | tubulin polymerization promoting protein |
| 0.00917 | 1.364 | 17.0 | 23.2 | MINK1 | misshapen-like kinase 1 |
| 0.00919 | 1.807 | 7.6 | 13.8 | PAK1 | p21 protein (Cdc42/Rac)-activated kinase 1 |
| 0.00928 | 0.782 | 18.0 | 14.1 | PRKD3 | protein kinase D3 |
| 0.00934 | 0.673 | 8.3 | 5.6 | TCF4 | transcription factor 4 |
| 0.00941 | 0.780 | 15.2 | 11.9 | UBA5 | ubiquitin-like modifier activating enzyme 5 |
| 0.00942 | 0.770 | 9.6 | 7.4 | NVL | nuclear VCP-like |
| 0.00948 | 0.697 | 9.1 | 6.3 | EXOSC8 | exosome component 8 |
| 0.00948 | 1.538 | 5.0 | 7.7 | FBXO2 | F-box protein 2 |
| 0.00948 | 1.322 | 30.6 | 40.5 | FKBP8 | FK506 binding protein 8, 38kDa |
| 0.00951 | 0.741 | 17.9 | 13.3 | LOC102724993 | nuclear pore complex-interacting protein family member A1-like |
| 0.00954 | 0.758 | 10.3 | 7.8 | LOC101927556 | uncharacterized LOC101927556 |
| 0.00962 | 0.702 | 19.6 | 13.7 | ZNF43 | zinc finger protein 43 |
| 0.00964 | 0.725 | 7.3 | 5.3 | PGBD1 | piggyBac transposable element derived 1 |
| 0.00965 | 2.126 | 14.4 | 30.5 | C1orf198 | chromosome 1 open reading frame 198 |
| 0.00968 | 1.332 | 15.1 | 20.1 | HEXA | hexosaminidase A (alpha polypeptide) |
| 0.00969 | 0.774 | 24.1 | 18.6 | TOP1 | topoisomerase (DNA) I |
| 0.00972 | 0.882 | 18.3 | 16.1 | KMT2D | lysine (K)-specific methyltransferase 2D |
| 0.00978 | 0.617 | 15.0 | 9.2 | MSI1 | musashi RNA-binding protein 1 |
| 0.00984 | 1.876 | 8.0 | 15.1 | KIAA0513 | KIAA0513 |
| 0.00984 | 1.637 | 5.6 | 9.2 | PARP12 | poly (ADP-ribose) polymerase family, member 12 |
| 0.00985 | 0.794 | 10.8 | 8.5 | INTS6 | integrator complex subunit 6 |
| 0.00985 | 1.318 | 9.2 | 12.1 | SNX18 | sorting nexin 18 |
| 0.00989 | 1.470 | 29.8 | 43.9 | CD164 | CD164 molecule, sialomucin |
| 0.00990 | 1.426 | 7.0 | 10.0 | MPP1 | membrane protein, palmitoylated 1, 55kDa |
| 0.00997 | 0.837 | 14.6 | 12.2 | PPIP5K2 | diphosphoinositol pentakisphosphate kinase 2 |
| 0.01001 | 0.700 | 22.7 | 15.9 | KIAA0754 | KIAA0754 |
| 0.01002 | 0.629 | 10.4 | 6.5 | SLC24A5 | solute carrier family 24 (sodium/potassium/calcium exchanger), member 5 |
| 0.01008 | 0.810 | 45.6 | 36.9 | ZC3H11A | zinc finger CCCH-type containing 11A |
| 0.01008 | 1.555 | 13.7 | 21.3 | ALDH2 | aldehyde dehydrogenase 2 family (mitochondrial) |
| 0.01011 | 2.594 | 5.8 | 15.1 | MAP7 | microtubule-associated protein 7 |
| 0.01017 | 0.723 | 16.6 | 12.0 | FKBP1AP1 | FK506 binding protein 1A, 12kDa pseudogene 1 |
| 0.01023 | 3.700 | 13.2 | 48.9 | CERCAM | cerebral endothelial cell adhesion molecule |
| 0.01026 | 0.765 | 14.7 | 11.3 | ZCCHC8 | zinc finger, CCHC domain containing 8 |
| 0.01027 | 1.317 | 5.0 | 6.6 | PPFIBP2 | PTPRF interacting protein, binding protein 2 (liprin beta 2) |
| 0.01036 | 0.570 | 12.5 | 7.1 | ZNF649 | zinc finger protein 649 |
| 0.01048 | 1.402 | 9.1 | 12.7 | RRM2B | ribonucleotide reductase M2 B (TP53 inducible) |
| 0.01052 | 2.251 | 5.0 | 11.3 | MX2 | myxovirus (influenza virus) resistance 2 (mouse) |
| 0.01052 | 1.513 | 5.8 | 8.8 | LYRM1 | LYR motif containing 1 |
| 0.01059 | 1.314 | 11.6 | 15.3 | BECN1 | beclin 1, autophagy related |
| 0.01063 | 2.648 | 5.5 | 14.5 | RAB40B | RAB40B, member RAS oncogene family |
| 0.01064 | 2.668 | 6.3 | 16.8 | PRKCB | protein kinase C, beta |
| 0.01065 | 2.149 | 6.4 | 13.8 | ARRDC2 | arrestin domain containing 2 |
| 0.01065 | 2.801 | 453.9 | 1271.3 | BCYRN1 | brain cytoplasmic RNA 1 |
| 0.01066 | 2.049 | 8.5 | 17.4 | PAQR8 | progestin and adipoQ receptor family member VIII |
| 0.01069 | 0.667 | 168.0 | 112.1 | SFPQ | splicing factor proline/glutamine-rich |
| 0.01070 | 0.667 | 24.3 | 16.2 | DMWD | dystrophia myotonica, WD repeat containing |
| 0.01077 | 1.759 | 25.8 | 45.4 | TRIM2 | tripartite motif containing 2 |
| 0.01083 | 0.776 | 28.1 | 21.8 | U2AF2 | U2 small nuclear RNA auxiliary factor 2 |
| 0.01089 | 0.636 | 23.6 | 15.0 | SPRED2 | sprouty-related, EVH1 domain containing 2 |
| 0.01097 | 0.770 | 9.4 | 7.2 | ZNF629 | zinc finger protein 629 |
| 0.01099 | 0.752 | 14.2 | 10.7 | GRAMD1A | GRAM domain containing 1A |
| 0.01105 | 0.838 | 6.0 | 5.0 | AC006115.3 | uncharacterized LOC101929059 |
| 0.01105 | 1.616 | 8.6 | 13.8 | TOLLIP | toll interacting protein |
| 0.01106 | 1.281 | 10.5 | 13.4 | TVP23B | trans-golgi network vesicle protein 23 homolog B (S. cerevisiae) |
| 0.01109 | 0.827 | 9.6 | 8.0 | SAP130 | Sin3A-associated protein, 130kDa |
| 0.01117 | 1.638 | 8.1 | 13.3 | NAT8L | N-acetyltransferase 8-like (GCN5-related, putative) |
| 0.01120 | 0.676 | 11.8 | 8.0 | ZNF134 | zinc finger protein 134 |
| 0.01120 | 1.286 | 7.7 | 9.9 | UFL1 | UFM1-specific ligase 1 |
| 0.01123 | 0.866 | 6.0 | 5.2 | PMF1-BGLAP | PMF1-BGLAP readthrough |
| 0.01126 | 0.804 | 7.3 | 5.9 | TIMM8A | translocase of inner mitochondrial membrane 8 homolog A (yeast) |
| 0.01136 | 0.720 | 12.8 | 9.2 | ZNF227 | zinc finger protein 227 |
| 0.01137 | 0.649 | 9.0 | 5.8 | SOX12 | SRY (sex determining region Y)-box 12 |
| 0.01142 | 3.318 | 10.3 | 34.3 | OLFM1 | olfactomedin 1 |
| 0.01144 | 0.752 | 9.4 | 7.1 | ADAMTS10 | ADAM metallopeptidase with thrombospondin type 1 motif, 10 |
| 0.01147 | 0.465 | 31.4 | 14.6 | SPRY4 | sprouty homolog 4 (Drosophila) |
| 0.01148 | 1.359 | 5.4 | 7.3 | PLA2G15 | phospholipase A2, group XV |
| 0.01149 | 0.761 | 12.3 | 9.4 | ZNF542P | zinc finger protein 542, pseudogene |
| 0.01150 | 2.964 | 5.5 | 16.3 | IFIT1 | interferon-induced protein with tetratricopeptide repeats 1 |
| 0.01152 | 0.700 | 9.1 | 6.4 | ARHGAP42 | Rho GTPase activating protein 42 |
| 0.01154 | 0.750 | 8.8 | 6.6 | ZNF568 | zinc finger protein 568 |
| 0.01154 | 0.760 | 7.8 | 5.9 | TOP3B | topoisomerase (DNA) III beta |
| 0.01161 | 0.779 | 24.3 | 18.9 | TAOK3 | TAO kinase 3 |
| 0.01161 | 0.630 | 26.2 | 16.5 | LOC102725293 | uncharacterized LOC102725293 |
| 0.01165 | 0.753 | 19.4 | 14.6 | FLVCR1 | feline leukemia virus subgroup C cellular receptor 1 |
| 0.01165 | 1.369 | 10.0 | 13.6 | IRF2 | interferon regulatory factor 2 |
| 0.01179 | 1.668 | 5.8 | 9.7 | TPD52L1 | tumor protein D52-like 1 |
| 0.01181 | 2.719 | 5.0 | 13.6 | SIGLEC1 | sialic acid binding Ig-like lectin 1, sialoadhesin |
| 0.01185 | 1.406 | 7.9 | 11.1 | KIAA1279 | KIAA1279 |
| 0.01188 | 2.165 | 11.4 | 24.6 | TMEM63A | transmembrane protein 63A |
| 0.01191 | 0.614 | 11.4 | 7.0 | TMEM39B | transmembrane protein 39B |
| 0.01193 | 0.744 | 28.5 | 21.2 | PPHLN1 | periphilin 1 |
| 0.01196 | 0.712 | 10.5 | 7.4 | KDM6B | lysine (K)-specific demethylase 6B |
| 0.01201 | 0.679 | 32.0 | 21.7 | APEX1 | APEX nuclease (multifunctional DNA repair enzyme) 1 |
| 0.01202 | 0.728 | 35.8 | 26.1 | IPO9 | importin 9 |
| 0.01207 | 0.588 | 11.1 | 6.5 | LOC100505502 | putative UPF0607 protein FLJ37424-like |
| 0.01213 | 0.774 | 8.1 | 6.3 | SLC35D1 | solute carrier family 35 (UDP-GlcA/UDP-GalNAc transporter), member D1 |
| 0.01216 | 1.786 | 6.8 | 12.1 | TBC1D12 | TBC1 domain family, member 12 |
| 0.01226 | 1.402 | 46.6 | 65.3 | PPT1 | palmitoyl-protein thioesterase 1 |
| 0.01232 | 1.258 | 22.6 | 28.4 | PFDN1 | prefoldin subunit 1 |
| 0.01237 | 0.702 | 13.6 | 9.6 | ZNF566 | zinc finger protein 566 |
| 0.01242 | 1.323 | 35.7 | 47.3 | SOD1 | superoxide dismutase 1, soluble |
| 0.01243 | 1.871 | 9.2 | 17.3 | NUPR1 | nuclear protein, transcriptional regulator, 1 |
| 0.01244 | 1.955 | 17.5 | 34.1 | MAN2A1 | mannosidase, alpha, class 2A, member 1 |
| 0.01248 | 3.378 | 6.4 | 21.7 | AATK | apoptosis-associated tyrosine kinase |
| 0.01251 | 0.718 | 10.8 | 7.7 | PARP2 | poly (ADP-ribose) polymerase 2 |
| 0.01252 | 1.214 | 9.2 | 11.2 | UBLCP1 | ubiquitin-like domain containing CTD phosphatase 1 |
| 0.01252 | 1.411 | 15.6 | 22.0 | NPTN | neuroplastin |
| 0.01255 | 1.666 | 7.4 | 12.3 | OSTF1 | osteoclast stimulating factor 1 |
| 0.01257 | 0.774 | 11.3 | 8.7 | MBD5 | methyl-CpG binding domain protein 5 |
| 0.01265 | 1.989 | 5.8 | 11.6 | REPS2 | RALBP1 associated Eps domain containing 2 |
| 0.01270 | 1.315 | 29.6 | 38.9 | NFE2L1 | nuclear factor, erythroid 2-like 1 |
| 0.01275 | 1.381 | 12.6 | 17.4 | PURA | purine-rich element binding protein A |
| 0.01276 | 1.648 | 31.0 | 51.1 | ASAH1 | N-acylsphingosine amidohydrolase (acid ceramidase) 1 |
| 0.01288 | 1.894 | 6.8 | 12.8 | MAST3 | microtubule associated serine/threonine kinase 3 |
| 0.01290 | 0.677 | 17.0 | 11.5 | ZNF253 | zinc finger protein 253 |
| 0.01296 | 1.360 | 5.9 | 8.1 | MYLIP | myosin regulatory light chain interacting protein |
| 0.01301 | 2.406 | 5.2 | 12.4 | PEX5L | peroxisomal biogenesis factor 5-like |
| 0.01301 | 1.357 | 11.2 | 15.2 | KLF13 | Kruppel-like factor 13 |
| 0.01305 | 1.397 | 6.7 | 9.3 | GNAZ | guanine nucleotide binding protein (G protein), alpha z polypeptide |
| 0.01313 | 0.615 | 141.9 | 87.2 | DBI | diazepam binding inhibitor (GABA receptor modulator, acyl-CoA binding protein) |
| 0.01314 | 0.869 | 5.9 | 5.1 | TAF4 | TAF4 RNA polymerase II, TATA box binding protein (TBP)-associated factor, 135kDa |
| 0.01321 | 0.525 | 30.2 | 15.9 | FOXG1 | forkhead box G1 |
| 0.01324 | 0.780 | 11.4 | 8.9 | NPIPA2 | nuclear pore complex interacting protein family, member A2 |
| 0.01330 | 0.765 | 7.6 | 5.8 | PCGF2 | polycomb group ring finger 2 |
| 0.01342 | 1.267 | 13.3 | 16.8 | TTC1 | tetratricopeptide repeat domain 1 |
| 0.01342 | 1.621 | 17.1 | 27.7 | MBNL2 | muscleblind-like splicing regulator 2 |
| 0.01344 | 0.820 | 7.4 | 6.1 | SETD1B | SET domain containing 1B |
| 0.01348 | 0.755 | 9.7 | 7.3 | ZNF595 | zinc finger protein 595 |
| 0.01350 | 1.962 | 5.4 | 10.6 | SLC22A15 | solute carrier family 22, member 15 |
| 0.01350 | 1.323 | 16.0 | 21.2 | GNPTG | N-acetylglucosamine-1-phosphate transferase, gamma subunit |
| 0.01352 | 1.459 | 7.6 | 11.1 | IGIP | IgA-inducing protein |
| 0.01354 | 0.732 | 16.0 | 11.7 | LINC01004 | long intergenic non-protein coding RNA 1004 |
| 0.01354 | 0.474 | 24.4 | 11.6 | SEMA6D | sema domain, transmembrane domain (TM), and cytoplasmic domain, (semaphorin) 6D |
| 0.01354 | 0.751 | 7.1 | 5.3 | ZNF571 | zinc finger protein 571 |
| 0.01359 | 0.815 | 10.7 | 8.7 | PIAS3 | protein inhibitor of activated STAT, 3 |
| 0.01360 | 0.754 | 21.1 | 15.9 | RBM4B | RNA binding motif protein 4B |
| 0.01364 | 1.567 | 8.5 | 13.4 | SCPEP1 | serine carboxypeptidase 1 |
| 0.01367 | 1.189 | 6.1 | 7.2 | ARHGEF18 | Rho/Rac guanine nucleotide exchange factor (GEF) 18 |
| 0.01368 | 0.787 | 9.5 | 7.5 | BCL9 | B-cell CLL/lymphoma 9 |
| 0.01370 | 3.144 | 6.3 | 19.8 | SLC24A2 | solute carrier family 24 (sodium/potassium/calcium exchanger), member 2 |
| 0.01372 | 1.429 | 6.4 | 9.2 | MANBA | mannosidase, beta A, lysosomal |
| 0.01380 | 2.894 | 29.8 | 86.1 | IFI44L | interferon-induced protein 44-like |
| 0.01390 | 0.728 | 9.0 | 6.5 | ZNF671 | zinc finger protein 671 |
| 0.01392 | 1.265 | 5.1 | 6.4 | ARHGAP25 | Rho GTPase activating protein 25 |
| 0.01392 | 1.448 | 27.2 | 39.3 | ATP6V1B2 | ATPase, H+ transporting, lysosomal 56/58kDa, V1 subunit B2 |
| 0.01395 | 2.555 | 11.8 | 30.1 | QDPR | quinoid dihydropteridine reductase |
| 0.01397 | 1.335 | 8.1 | 10.8 | ARHGEF10 | Rho guanine nucleotide exchange factor (GEF) 10 |
| 0.01398 | 0.756 | 10.8 | 8.1 | CHERP | calcium homeostasis endoplasmic reticulum protein |
| 0.01405 | 1.570 | 7.7 | 12.1 | LOC101929385 | uncharacterized LOC101929385 |
| 0.01406 | 1.331 | 27.6 | 36.7 | PAIP2 | poly(A) binding protein interacting protein 2 |
| 0.01407 | 1.589 | 5.8 | 9.3 | NUAK1 | NUAK family, SNF1-like kinase, 1 |
| 0.01413 | 1.686 | 14.9 | 25.2 | FAM102A | family with sequence similarity 102, member A |
| 0.01420 | 0.692 | 8.3 | 5.7 | TAS2R14 | taste receptor, type 2, member 14 |
| 0.01422 | 1.393 | 15.9 | 22.1 | RNF141 | ring finger protein 141 |
| 0.01422 | 1.321 | 8.6 | 11.4 | OGFR | opioid growth factor receptor |
| 0.01424 | 0.835 | 21.4 | 17.9 | PPP6R2 | protein phosphatase 6, regulatory subunit 2 |
| 0.01426 | 0.756 | 12.6 | 9.5 | FAM222B | family with sequence similarity 222, member B |
| 0.01427 | 0.535 | 12.2 | 6.5 | ZIC1 | Zic family member 1 |
| 0.01434 | 0.675 | 17.2 | 11.6 | LOC101926943 | uncharacterized LOC101926943 |
| 0.01434 | 1.647 | 13.5 | 22.2 | APBB1 | amyloid beta (A4) precursor protein-binding, family B, member 1 (Fe65) |
| 0.01436 | 0.696 | 21.5 | 15.0 | MTA1 | metastasis associated 1 |
| 0.01439 | 1.521 | 14.0 | 21.3 | FNBP1 | formin binding protein 1 |
| 0.01440 | 1.860 | 6.1 | 11.4 | COL4A5 | collagen, type IV, alpha 5 |
| 0.01451 | 0.678 | 18.3 | 12.4 | ZNF431 | zinc finger protein 431 |
| 0.01457 | 1.680 | 44.0 | 73.9 | DKK3 | dickkopf WNT signaling pathway inhibitor 3 |
| 0.01458 | 1.648 | 17.2 | 28.4 | PARP9 | poly (ADP-ribose) polymerase family, member 9 |
| 0.01460 | 1.442 | 8.2 | 11.8 | SPRYD3 | SPRY domain containing 3 |
| 0.01465 | 0.908 | 5.8 | 5.2 | TTC27 | tetratricopeptide repeat domain 27 |
| 0.01469 | 1.294 | 6.7 | 8.6 | FAM160B1 | family with sequence similarity 160, member B1 |
| 0.01470 | 0.776 | 15.4 | 11.9 | PIBF1 | progesterone immunomodulatory binding factor 1 |
| 0.01472 | 0.524 | 16.3 | 8.5 | HIST1H2AJ | histone cluster 1, H2aj |
| 0.01476 | 0.743 | 17.2 | 12.8 | TUBGCP4 | tubulin, gamma complex associated protein 4 |
| 0.01479 | 0.724 | 7.6 | 5.5 | DOCK6 | dedicator of cytokinesis 6 |
| 0.01496 | 2.220 | 31.7 | 70.3 | PLEKHB1 | pleckstrin homology domain containing, family B (evectins) member 1 |
| 0.01502 | 0.788 | 52.9 | 41.7 | HNRNPL | heterogeneous nuclear ribonucleoprotein L |
| 0.01507 | 1.434 | 29.5 | 42.4 | PLD3 | phospholipase D family, member 3 |
| 0.01509 | 0.655 | 16.8 | 11.0 | ZNF92 | zinc finger protein 92 |
| 0.01512 | 1.378 | 13.6 | 18.8 | RGL1 | ral guanine nucleotide dissociation stimulator-like 1 |
| 0.01514 | 0.679 | 8.5 | 5.7 | LINC01089 | long intergenic non-protein coding RNA 1089 |
| 0.01517 | 0.640 | 26.3 | 16.8 | SACS | sacsin molecular chaperone |
| 0.01523 | 2.433 | 22.9 | 55.7 | ATP1B1 | ATPase, Na+/K+ transporting, beta 1 polypeptide |
| 0.01530 | 0.820 | 6.5 | 5.4 | DCBLD1 | discoidin, CUB and LCCL domain containing 1 |
| 0.01532 | 1.436 | 20.2 | 29.0 | VAT1 | vesicle amine transport 1 |
| 0.01540 | 0.812 | 16.4 | 13.3 | TP53BP2 | tumor protein p53 binding protein, 2 |
| 0.01540 | 3.868 | 6.5 | 25.2 | MYRF | myelin regulatory factor |
| 0.01541 | 1.299 | 11.8 | 15.3 | LPIN2 | lipin 2 |
| 0.01543 | 0.839 | 6.4 | 5.4 | PGP | phosphoglycolate phosphatase |
| 0.01545 | 3.011 | 15.6 | 46.8 | ABCA2 | ATP-binding cassette, sub-family A (ABC1), member 2 |
| 0.01545 | 0.581 | 16.0 | 9.3 | NLGN4X | neuroligin 4, X-linked |
| 0.01555 | 0.765 | 7.6 | 5.8 | C1orf56 | chromosome 1 open reading frame 56 |
| 0.01556 | 1.747 | 31.7 | 55.4 | LGALS3BP | lectin, galactoside-binding, soluble, 3 binding protein |
| 0.01556 | 4.930 | 93.5 | 461.1 | PLP1 | proteolipid protein 1 |
| 0.01558 | 1.738 | 5.3 | 9.3 | IFIH1 | interferon induced with helicase C domain 1 |
| 0.01559 | 1.537 | 17.5 | 27.0 | SCOC | short coiled-coil protein |
| 0.01562 | 1.382 | 7.5 | 10.3 | SLC41A3 | solute carrier family 41, member 3 |
| 0.01562 | 0.775 | 17.8 | 13.8 | RSPRY1 | ring finger and SPRY domain containing 1 |
| 0.01571 | 1.496 | 7.8 | 11.6 | GCLC | glutamate-cysteine ligase, catalytic subunit |
| 0.01572 | 0.505 | 13.9 | 7.0 | MAP3K1 | mitogen-activated protein kinase kinase kinase 1, E3 ubiquitin protein ligase |
| 0.01573 | 0.633 | 9.4 | 5.9 | FAM84A | family with sequence similarity 84, member A |
| 0.01578 | 1.967 | 6.9 | 13.6 | KCNAB2 | potassium voltage-gated channel, shaker-related subfamily, beta member 2 |
| 0.01579 | 4.587 | 7.9 | 36.2 | ERMN | ermin, ERM-like protein |
| 0.01581 | 1.266 | 23.2 | 29.3 | EFCAB14 | EF-hand calcium binding domain 14 |
| 0.01587 | 1.915 | 21.2 | 40.6 | KIF1A | kinesin family member 1A |
| 0.01609 | 0.611 | 28.4 | 17.4 | LOC102725374 | uncharacterized LOC102725374 |
| 0.01609 | 1.456 | 9.9 | 14.4 | IPO13 | importin 13 |
| 0.01616 | 2.090 | 21.0 | 44.0 | CTSZ | cathepsin Z |
| 0.01618 | 0.818 | 12.0 | 9.8 | MAP4K3 | mitogen-activated protein kinase kinase kinase kinase 3 |
| 0.01624 | 0.792 | 36.4 | 28.8 | CS | citrate synthase |
| 0.01626 | 0.731 | 11.6 | 8.5 | RNF214 | ring finger protein 214 |
| 0.01629 | 1.910 | 10.7 | 20.4 | COLEC12 | collectin sub-family member 12 |
| 0.01630 | 2.317 | 18.9 | 43.7 | KIAA1598 | KIAA1598 |
| 0.01632 | 1.162 | 13.9 | 16.2 | AKAP10 | A kinase (PRKA) anchor protein 10 |
| 0.01633 | 0.802 | 23.2 | 18.6 | RBM23 | RNA binding motif protein 23 |
| 0.01637 | 1.533 | 28.5 | 43.7 | RNF213 | ring finger protein 213 |
| 0.01646 | 1.636 | 42.5 | 69.5 | YWHAH | tyrosine 3-monooxygenase/tryptophan 5-monooxygenase activation protein, eta |
| 0.01652 | 0.594 | 27.0 | 16.0 | TRIM24 | tripartite motif containing 24 |
| 0.01653 | 1.599 | 5.4 | 8.6 | TMEM98 | transmembrane protein 98 |
| 0.01655 | 1.417 | 7.6 | 10.8 | PLEKHA1 | pleckstrin homology domain containing, family A (phosphoinositide binding specific) member 1 |
| 0.01658 | 0.633 | 20.6 | 13.1 | PLXDC1 | plexin domain containing 1 |
| 0.01660 | 0.826 | 48.1 | 39.7 | PRRC2C | proline-rich coiled-coil 2C |
| 0.01662 | 0.782 | 10.8 | 8.5 | WIZ | widely interspaced zinc finger motifs |
| 0.01666 | 1.327 | 14.3 | 19.0 | PPP1R2 | protein phosphatase 1, regulatory (inhibitor) subunit 2 |
| 0.01669 | 1.370 | 13.4 | 18.3 | PPP3CB | protein phosphatase 3, catalytic subunit, beta isozyme |
| 0.01670 | 0.807 | 9.3 | 7.5 | MAPKBP1 | mitogen-activated protein kinase binding protein 1 |
| 0.01672 | 0.702 | 9.8 | 6.9 | RAD54B | RAD54 homolog B (S. cerevisiae) |
| 0.01673 | 2.668 | 5.3 | 14.0 | CNTNAP4 | contactin associated protein-like 4 |
| 0.01698 | 1.655 | 6.7 | 11.1 | DDX58 | DEAD (Asp-Glu-Ala-Asp) box polypeptide 58 |
| 0.01703 | 0.807 | 6.6 | 5.3 | ABCB10 | ATP-binding cassette, sub-family B (MDR/TAP), member 10 |
| 0.01705 | 1.515 | 22.5 | 34.0 | BMP2K | BMP2 inducible kinase |
| 0.01708 | 2.343 | 18.0 | 42.1 | GALNT15 | polypeptide N-acetylgalactosaminyltransferase 15 |
| 0.01709 | 0.745 | 7.0 | 5.2 | OSGEPL1-AS1 | OSGEPL1 antisense RNA 1 |
| 0.01710 | 1.403 | 51.7 | 72.6 | ARHGAP21 | Rho GTPase activating protein 21 |
| 0.01719 | 0.871 | 11.8 | 10.3 | COG3 | component of oligomeric golgi complex 3 |
| 0.01721 | 0.667 | 13.3 | 8.9 | NLGN1 | neuroligin 1 |
| 0.01727 | 3.588 | 7.7 | 27.7 | CNTN2 | contactin 2 (axonal) |
| 0.01727 | 0.693 | 8.1 | 5.6 | LOC102725171 | uncharacterized LOC102725171 |
| 0.01735 | 0.769 | 19.2 | 14.8 | PHF12 | PHD finger protein 12 |
| 0.01750 | 1.412 | 7.7 | 10.8 | FAM228B | family with sequence similarity 228, member B |
| 0.01763 | 1.342 | 9.5 | 12.7 | CREBRF | CREB3 regulatory factor |
| 0.01770 | 0.701 | 16.1 | 11.3 | FBL | fibrillarin |
| 0.01776 | 0.768 | 7.7 | 5.9 | NOX4 | NADPH oxidase 4 |
| 0.01778 | 0.790 | 25.7 | 20.3 | YLPM1 | YLP motif containing 1 |
| 0.01778 | 0.785 | 8.7 | 6.8 | PCID2 | PCI domain containing 2 |
| 0.01779 | 2.863 | 5.1 | 14.5 | SPOCK3 | sparc/osteonectin, cwcv and kazal-like domains proteoglycan (testican) 3 |
| 0.01782 | 1.379 | 5.4 | 7.5 | CADM3-AS1 | CADM3 antisense RNA 1 |
| 0.01792 | 0.742 | 9.3 | 6.9 | N4BP2 | NEDD4 binding protein 2 |
| 0.01794 | 2.066 | 7.5 | 15.4 | SLC31A2 | solute carrier family 31 (copper transporter), member 2 |
| 0.01804 | 0.782 | 31.0 | 24.2 | ADNP | activity-dependent neuroprotector homeobox |
| 0.01806 | 0.759 | 7.8 | 5.9 | ZNF23 | zinc finger protein 23 |
| 0.01807 | 1.402 | 6.9 | 9.7 | DDB2 | damage-specific DNA binding protein 2, 48kDa |
| 0.01814 | 1.705 | 5.6 | 9.5 | PRIMA1 | proline rich membrane anchor 1 |
| 0.01815 | 0.758 | 9.7 | 7.4 | ZNF17 | zinc finger protein 17 |
| 0.01823 | 0.736 | 11.1 | 8.2 | CTB-118N6.3 | uncharacterized LOC101927233 |
| 0.01824 | 1.560 | 5.2 | 8.1 | GOT1 | glutamic-oxaloacetic transaminase 1, soluble |
| 0.01825 | 0.735 | 23.9 | 17.5 | HIP1 | huntingtin interacting protein 1 |
| 0.01827 | 0.554 | 13.9 | 7.7 | KLF12 | Kruppel-like factor 12 |
| 0.01827 | 1.360 | 6.6 | 9.0 | C1GALT1 | core 1 synthase, glycoprotein-N-acetylgalactosamine 3-beta-galactosyltransferase, 1 |
| 0.01832 | 0.764 | 11.4 | 8.7 | TDRD3 | tudor domain containing 3 |
| 0.01843 | 1.556 | 20.1 | 31.3 | WARS | tryptophanyl-tRNA synthetase |
| 0.01848 | 1.511 | 7.8 | 11.9 | PGAM4 | phosphoglycerate mutase family member 4 |
| 0.01851 | 1.751 | 5.3 | 9.3 | KLHL32 | kelch-like family member 32 |
| 0.01853 | 1.949 | 5.1 | 9.9 | LOC101926887 | uncharacterized LOC101926887 |
| 0.01858 | 1.453 | 92754.9 | 134760.4 | RN7SL2 | RNA, 7SL, cytoplasmic 2 |
| 0.01858 | 2.781 | 25.4 | 70.6 | TUBB4A | tubulin, beta 4A class IVa |
| 0.01859 | 1.554 | 5.9 | 9.2 | C2orf74 | chromosome 2 open reading frame 74 |
| 0.01863 | 1.316 | 34.7 | 45.6 | FAM127A | family with sequence similarity 127, member A |
| 0.01868 | 1.480 | 5.9 | 8.8 | SLC9A9 | solute carrier family 9, subfamily A (NHE9, cation proton antiporter 9), member 9 |
| 0.01870 | 2.652 | 8.2 | 21.6 | RAPGEF5 | Rap guanine nucleotide exchange factor (GEF) 5 |
| 0.01873 | 0.677 | 8.2 | 5.6 | NOSTRIN | nitric oxide synthase trafficking |
| 0.01874 | 3.179 | 14.2 | 45.3 | SEP4 | septin 4 |
| 0.01879 | 1.436 | 32.4 | 46.6 | PGAM1 | phosphoglycerate mutase 1 (brain) |
| 0.01886 | 1.207 | 64.5 | 77.9 | MORF4L1 | mortality factor 4 like 1 |
| 0.01888 | 1.215 | 5.5 | 6.7 | RPS6KA4 | ribosomal protein S6 kinase, 90kDa, polypeptide 4 |
| 0.01888 | 1.715 | 8.1 | 13.8 | MRPL41 | mitochondrial ribosomal protein L41 |
| 0.01892 | 0.779 | 73.2 | 57.0 | HNRNPU | heterogeneous nuclear ribonucleoprotein U (scaffold attachment factor A) |
| 0.01896 | 1.580 | 6.5 | 10.2 | NDFIP2 | Nedd4 family interacting protein 2 |
| 0.01908 | 0.792 | 11.5 | 9.1 | POLR1A | polymerase (RNA) I polypeptide A, 194kDa |
| 0.01908 | 0.791 | 23.8 | 18.8 | HNRNPA1L2 | heterogeneous nuclear ribonucleoprotein A1-like 2 |
| 0.01909 | 1.288 | 18.5 | 23.9 | ISCU | iron-sulfur cluster assembly enzyme |
| 0.01910 | 1.338 | 6.5 | 8.7 | ST3GAL4 | ST3 beta-galactoside alpha-2,3-sialyltransferase 4 |
| 0.01913 | 0.713 | 14.8 | 10.6 | RP11-20B24.5 | uncharacterized LOC101927018 |
| 0.01915 | 1.497 | 13.5 | 20.3 | GAA | glucosidase, alpha; acid |
| 0.01918 | 1.242 | 19.1 | 23.8 | M6PR | mannose-6-phosphate receptor (cation dependent) |
| 0.01921 | 1.365 | 5.6 | 7.7 | IFIT5 | interferon-induced protein with tetratricopeptide repeats 5 |
| 0.01928 | 2.005 | 10.4 | 20.8 | ARRB1 | arrestin, beta 1 |
| 0.01932 | 2.410 | 26.0 | 62.7 | CNP | 2',3'-cyclic nucleotide 3' phosphodiesterase |
| 0.01932 | 0.842 | 25.1 | 21.1 | UFD1L | ubiquitin fusion degradation 1 like (yeast) |
| 0.01949 | 1.330 | 23.6 | 31.4 | SLC11A2 | solute carrier family 11 (proton-coupled divalent metal ion transporter), member 2 |
| 0.01954 | 3.303 | 38.4 | 126.7 | IFI6 | interferon, alpha-inducible protein 6 |
| 0.01963 | 1.403 | 14.9 | 20.9 | CNDP2 | CNDP dipeptidase 2 (metallopeptidase M20 family) |
| 0.01965 | 2.044 | 5.3 | 10.9 | SCN1B | sodium channel, voltage-gated, type I, beta subunit |
| 0.01967 | 1.647 | 5.8 | 9.5 | DDX60 | DEAD (Asp-Glu-Ala-Asp) box polypeptide 60 |
| 0.01969 | 0.724 | 24.7 | 17.9 | ZNF417 | zinc finger protein 417 |
| 0.01969 | 1.340 | 5.4 | 7.3 | PRKCD | protein kinase C, delta |
| 0.01971 | 1.220 | 5.0 | 6.1 | ITGAL | integrin, alpha L (antigen CD11A (p180), lymphocyte function-associated antigen 1; alpha polypeptide) |
| 0.01973 | 0.792 | 6.7 | 5.3 | ICA1 | islet cell autoantigen 1, 69kDa |
| 0.01975 | 0.757 | 21.6 | 16.4 | ZNF608 | zinc finger protein 608 |
| 0.01991 | 0.691 | 7.5 | 5.2 | LOC102724133 | uncharacterized LOC102724133 |
| 0.01992 | 0.803 | 21.4 | 17.2 | MAPK1IP1L | mitogen-activated protein kinase 1 interacting protein 1-like |
| 0.01995 | 1.993 | 7.4 | 14.7 | DLG2 | discs, large homolog 2 (Drosophila) |
| 0.01999 | 0.615 | 11.7 | 7.2 | ESAM | endothelial cell adhesion molecule |
| 0.02006 | 1.289 | 5.1 | 6.5 | R3HCC1L | R3H domain and coiled-coil containing 1-like |
| 0.02027 | 0.829 | 10.3 | 8.5 | FBXW8 | F-box and WD repeat domain containing 8 |
| 0.02030 | 0.763 | 11.5 | 8.8 | ZNF234 | zinc finger protein 234 |
| 0.02030 | 1.891 | 8.2 | 15.4 | LILRB4 | leukocyte immunoglobulin-like receptor, subfamily B (with TM and ITIM domains), member 4 |
| 0.02034 | 1.433 | 14.7 | 21.1 | DTX3L | deltex 3 like, E3 ubiquitin ligase |
| 0.02037 | 1.493 | 9.0 | 13.5 | B3GNT1 | UDP-GlcNAc:betaGal beta-1,3-N-acetylglucosaminyltransferase 1 |
| 0.02043 | 1.365 | 14.8 | 20.2 | TMED7 | transmembrane emp24 protein transport domain containing 7 |
| 0.02044 | 2.084 | 40.7 | 84.7 | GSN | gelsolin |
| 0.02047 | 0.764 | 11.7 | 8.9 | CAPN15 | calpain 15 |
| 0.02056 | 1.463 | 8.8 | 12.8 | HPS3 | Hermansky-Pudlak syndrome 3 |
| 0.02058 | 0.544 | 23.7 | 12.9 | SCARNA18 | small Cajal body-specific RNA 18 |
| 0.02060 | 1.438 | 53.0 | 76.2 | SERINC1 | serine incorporator 1 |
| 0.02066 | 2.155 | 5.2 | 11.2 | CMPK2 | cytidine monophosphate (UMP-CMP) kinase 2, mitochondrial |
| 0.02068 | 0.830 | 12.8 | 10.7 | VPS36 | vacuolar protein sorting 36 homolog (S. cerevisiae) |
| 0.02077 | 0.524 | 12.0 | 6.3 | FAM27E3 | family with sequence similarity 27, member E3 |
| 0.02082 | 0.849 | 15.9 | 13.5 | EP400 | E1A binding protein p400 |
| 0.02083 | 1.964 | 18.5 | 36.4 | LIPA | lipase A, lysosomal acid, cholesterol esterase |
| 0.02089 | 0.492 | 41.5 | 20.4 | ETV1 | ets variant 1 |
| 0.02091 | 1.445 | 8.6 | 12.4 | ATP6V1H | ATPase, H+ transporting, lysosomal 50/57kDa, V1 subunit H |
| 0.02100 | 1.990 | 5.2 | 10.3 | HERC6 | HECT and RLD domain containing E3 ubiquitin protein ligase family member 6 |
| 0.02101 | 0.801 | 13.0 | 10.4 | DGCR8 | DGCR8 microprocessor complex subunit |
| 0.02101 | 2.243 | 6.4 | 14.3 | SRCIN1 | SRC kinase signaling inhibitor 1 |
| 0.02105 | 1.181 | 5.7 | 6.8 | BBS10 | Bardet-Biedl syndrome 10 |
| 0.02105 | 0.699 | 9.8 | 6.8 | LOC101928641 | zinc finger protein 816-like |
| 0.02105 | 1.977 | 10.7 | 21.1 | BEST1 | bestrophin 1 |
| 0.02110 | 0.728 | 39.3 | 28.6 | DDX39B | DEAD (Asp-Glu-Ala-Asp) box polypeptide 39B |
| 0.02115 | 3.742 | 15.3 | 57.4 | DBNDD2 | dysbindin (dystrobrevin binding protein 1) domain containing 2 |
| 0.02125 | 0.757 | 24.5 | 18.5 | ZNF528 | zinc finger protein 528 |
| 0.02130 | 0.660 | 9.8 | 6.5 | GAS5-AS1 | GAS5 antisense RNA 1 |
| 0.02144 | 0.658 | 13.8 | 9.1 | LOC101929191 | uncharacterized LOC101929191 |
| 0.02145 | 1.383 | 7.7 | 10.6 | F8A1 | coagulation factor VIII-associated 1 |
| 0.02145 | 0.841 | 24.6 | 20.7 | WDR33 | WD repeat domain 33 |
| 0.02147 | 0.483 | 27.5 | 13.3 | LRRN1 | leucine rich repeat neuronal 1 |
| 0.02147 | 1.387 | 5.0 | 7.0 | PLD1 | phospholipase D1, phosphatidylcholine-specific |
| 0.02148 | 1.299 | 6.7 | 8.7 | PQLC1 | PQ loop repeat containing 1 |
| 0.02150 | 1.783 | 22.3 | 39.7 | ANK2 | ankyrin 2, neuronal |
| 0.02153 | 0.780 | 10.5 | 8.2 | ZNF346 | zinc finger protein 346 |
| 0.02154 | 0.716 | 9.6 | 6.8 | ZNF432 | zinc finger protein 432 |
| 0.02159 | 0.723 | 11.8 | 8.6 | HEATR1 | HEAT repeat containing 1 |
| 0.02161 | 0.815 | 19.9 | 16.3 | ZNF841 | zinc finger protein 841 |
| 0.02166 | 0.800 | 21.3 | 17.0 | CCNH | cyclin H |
| 0.02169 | 2.517 | 6.3 | 16.0 | LHPP | phospholysine phosphohistidine inorganic pyrophosphate phosphatase |
| 0.02172 | 1.215 | 23.8 | 29.0 | PLEKHB2 | pleckstrin homology domain containing, family B (evectins) member 2 |
| 0.02186 | 0.825 | 6.5 | 5.3 | LYSMD1 | LysM, putative peptidoglycan-binding, domain containing 1 |
| 0.02188 | 1.560 | 47.0 | 73.4 | TPP1 | tripeptidyl peptidase I |
| 0.02189 | 0.774 | 11.9 | 9.2 | SF3B4 | splicing factor 3b, subunit 4, 49kDa |
| 0.02189 | 1.301 | 7.1 | 9.2 | SNX29 | sorting nexin 29 |
| 0.02191 | 0.552 | 11.9 | 6.6 | NETO2 | neuropilin (NRP) and tolloid (TLL)-like 2 |
| 0.02192 | 0.865 | 6.0 | 5.2 | MYNN | myoneurin |
| 0.02193 | 1.824 | 9.2 | 16.8 | OSBPL1A | oxysterol binding protein-like 1A |
| 0.02197 | 2.145 | 16.9 | 36.2 | CHN1 | chimerin 1 |
| 0.02201 | 1.586 | 5.7 | 9.1 | TNFSF13B | tumor necrosis factor (ligand) superfamily, member 13b |
| 0.02205 | 0.697 | 14.7 | 10.2 | CENPM | centromere protein M |
| 0.02206 | 2.945 | 11.8 | 34.6 | BCAS1 | breast carcinoma amplified sequence 1 |
| 0.02211 | 1.462 | 24.1 | 35.2 | LOC728392 | uncharacterized LOC728392 |
| 0.02215 | 3.059 | 7.8 | 23.8 | OAS2 | 2'-5'-oligoadenylate synthetase 2, 69/71kDa |
| 0.02216 | 0.599 | 10.2 | 6.1 | LCORL | ligand dependent nuclear receptor corepressor-like |
| 0.02219 | 2.266 | 6.5 | 14.8 | SLC6A6 | solute carrier family 6 (neurotransmitter transporter), member 6 |
| 0.02229 | 0.812 | 24.3 | 19.7 | CERS5 | ceramide synthase 5 |
| 0.02230 | 0.712 | 24.4 | 17.4 | MIRLET7BHG | MIRLET7B host gene (non-protein coding) |
| 0.02232 | 0.570 | 9.9 | 5.7 | FLJ22184 | putative uncharacterized protein FLJ22184 |
| 0.02238 | 0.717 | 27.2 | 19.5 | QSER1 | glutamine and serine rich 1 |
| 0.02243 | 0.858 | 12.2 | 10.4 | DIS3L2 | DIS3 like 3'-5' exoribonuclease 2 |
| 0.02251 | 1.533 | 13.3 | 20.4 | ADIPOR2 | adiponectin receptor 2 |
| 0.02256 | 1.517 | 13.2 | 20.0 | ELOVL1 | ELOVL fatty acid elongase 1 |
| 0.02257 | 0.527 | 18.2 | 9.6 | AC093642.3 | uncharacterized LOC102723927 |
| 0.02261 | 0.765 | 33.3 | 25.4 | PLCG1 | phospholipase C, gamma 1 |
| 0.02266 | 0.767 | 10.8 | 8.3 | CYB5D1 | cytochrome b5 domain containing 1 |
| 0.02283 | 1.375 | 46.8 | 64.3 | MDM2 | MDM2 oncogene, E3 ubiquitin protein ligase |
| 0.02284 | 1.182 | 5.4 | 6.4 | GALK2 | galactokinase 2 |
| 0.02289 | 1.344 | 8.2 | 11.1 | BLOC1S2 | biogenesis of lysosomal organelles complex-1, subunit 2 |
| 0.02294 | 0.773 | 26.3 | 20.3 | PTOV1 | prostate tumor overexpressed 1 |
| 0.02299 | 1.267 | 7.4 | 9.3 | RNF19B | ring finger protein 19B |
| 0.02300 | 0.810 | 20.3 | 16.4 | SMEK1 | SMEK homolog 1, suppressor of mek1 (Dictyostelium) |
| 0.02307 | 1.175 | 5.2 | 6.1 | VPS37C | vacuolar protein sorting 37 homolog C (S. cerevisiae) |
| 0.02309 | 0.764 | 7.7 | 5.9 | TBKBP1 | TBK1 binding protein 1 |
| 0.02321 | 1.817 | 5.7 | 10.4 | MAP6D1 | MAP6 domain containing 1 |
| 0.02331 | 0.527 | 42.5 | 22.4 | RHOJ | ras homolog family member J |
| 0.02331 | 0.753 | 12.6 | 9.5 | POU2F1 | POU class 2 homeobox 1 |
| 0.02331 | 1.214 | 12.4 | 15.0 | SNX24 | sorting nexin 24 |
| 0.02339 | 2.702 | 15.2 | 41.2 | PIP4K2A | phosphatidylinositol-5-phosphate 4-kinase, type II, alpha |
| 0.02340 | 0.780 | 10.1 | 7.9 | ZACN | zinc activated ligand-gated ion channel |
| 0.02342 | 0.649 | 8.1 | 5.3 | LOC101928927 | uncharacterized LOC101928927 |
| 0.02346 | 0.693 | 26.7 | 18.5 | NAA16 | N(alpha)-acetyltransferase 16, NatA auxiliary subunit |
| 0.02351 | 1.397 | 9.7 | 13.6 | STX4 | syntaxin 4 |
| 0.02353 | 1.398 | 15.1 | 21.1 | FDFT1 | farnesyl-diphosphate farnesyltransferase 1 |
| 0.02361 | 0.476 | 48.3 | 23.0 | FABP7 | fatty acid binding protein 7, brain |
| 0.02364 | 4.097 | 9.3 | 38.0 | TMEM144 | transmembrane protein 144 |
| 0.02370 | 0.639 | 11.3 | 7.2 | EPN2-AS1 | EPN2 antisense RNA 1 |
| 0.02374 | 2.845 | 13.7 | 38.9 | PLEKHH1 | pleckstrin homology domain containing, family H (with MyTH4 domain) member 1 |
| 0.02377 | 1.587 | 8.1 | 12.9 | KIF13B | kinesin family member 13B |
| 0.02399 | 0.658 | 9.3 | 6.1 | WDR86 | WD repeat domain 86 |
| 0.02400 | 0.798 | 19.6 | 15.6 | PHKB | phosphorylase kinase, beta |
| 0.02403 | 0.749 | 7.5 | 5.6 | GPR161 | G protein-coupled receptor 161 |
| 0.02404 | 1.250 | 9.8 | 12.2 | RAB7L1 | RAB7, member RAS oncogene family-like 1 |
| 0.02406 | 0.781 | 36.1 | 28.2 | IRGQ | immunity-related GTPase family, Q |
| 0.02407 | 0.500 | 20.9 | 10.4 | GRIA4 | glutamate receptor, ionotropic, AMPA 4 |
| 0.02413 | 1.781 | 5.6 | 10.0 | ROGDI | rogdi homolog (Drosophila) |
| 0.02436 | 0.657 | 80.6 | 53.0 | PON2 | paraoxonase 2 |
| 0.02436 | 1.528 | 8.8 | 13.4 | MOSPD2 | motile sperm domain containing 2 |
| 0.02442 | 0.687 | 19.5 | 13.4 | GPR125 | G protein-coupled receptor 125 |
| 0.02472 | 1.287 | 17.1 | 22.0 | TALDO1 | transaldolase 1 |
| 0.02475 | 0.892 | 52.8 | 47.1 | SF1 | splicing factor 1 |
| 0.02475 | 0.815 | 6.2 | 5.1 | CDC6 | cell division cycle 6 |
| 0.02479 | 0.705 | 17.6 | 12.4 | COG4 | component of oligomeric golgi complex 4 |
| 0.02480 | 1.362 | 13.6 | 18.5 | SCAMP2 | secretory carrier membrane protein 2 |
| 0.02484 | 0.664 | 15.7 | 10.4 | PLXNA2 | plexin A2 |
| 0.02485 | 2.139 | 5.0 | 10.7 | NPTX1 | neuronal pentraxin I |
| 0.02488 | 1.367 | 10.7 | 14.6 | CCPG1 | cell cycle progression 1 |
| 0.02488 | 0.672 | 13.2 | 8.9 | ZNF418 | zinc finger protein 418 |
| 0.02489 | 0.640 | 10.7 | 6.8 | HDAC2 | histone deacetylase 2 |
| 0.02494 | 0.830 | 6.1 | 5.1 | LOC102725334 | uncharacterized LOC102725334 |
| 0.02508 | 0.847 | 29.7 | 25.2 | BRD4 | bromodomain containing 4 |
| 0.02513 | 1.414 | 25.2 | 35.6 | PAQR3 | progestin and adipoQ receptor family member III |
| 0.02514 | 0.607 | 12.5 | 7.6 | ZNF711 | zinc finger protein 711 |
| 0.02524 | 1.509 | 5.7 | 8.6 | GNAI1 | guanine nucleotide binding protein (G protein), alpha inhibiting activity polypeptide 1 |
| 0.02530 | 0.853 | 22.4 | 19.1 | SPEN | spen family transcriptional repressor |
| 0.02537 | 1.434 | 20.5 | 29.4 | DLG1 | discs, large homolog 1 (Drosophila) |
| 0.02542 | 0.781 | 6.6 | 5.2 | TAS2R13 | taste receptor, type 2, member 13 |
| 0.02547 | 0.855 | 25.1 | 21.4 | SMEK2 | SMEK homolog 2, suppressor of mek1 (Dictyostelium) |
| 0.02548 | 0.874 | 10.5 | 9.1 | SART3 | squamous cell carcinoma antigen recognized by T cells 3 |
| 0.02550 | 2.307 | 21.5 | 49.6 | PRUNE2 | prune homolog 2 (Drosophila) |
| 0.02552 | 1.561 | 5.1 | 8.0 | MYO1D | myosin ID |
| 0.02554 | 1.274 | 10.8 | 13.7 | LNPEP | leucyl/cystinyl aminopeptidase |
| 0.02560 | 1.933 | 5.1 | 9.9 | EPSTI1 | epithelial stromal interaction 1 (breast) |
| 0.02566 | 0.597 | 14.4 | 8.6 | KDM5B | lysine (K)-specific demethylase 5B |
| 0.02576 | 0.589 | 20.3 | 11.9 | PGAP1 | post-GPI attachment to proteins 1 |
| 0.02583 | 2.179 | 10.2 | 22.3 | DOCK5 | dedicator of cytokinesis 5 |
| 0.02587 | 1.256 | 19.2 | 24.2 | HIPK3 | homeodomain interacting protein kinase 3 |
| 0.02587 | 1.323 | 17.1 | 22.6 | PPA1 | pyrophosphatase (inorganic) 1 |
| 0.02588 | 1.490 | 17.6 | 26.2 | GM2A | GM2 ganglioside activator |
| 0.02598 | 0.677 | 13.0 | 8.8 | RAVER1 | ribonucleoprotein, PTB-binding 1 |
| 0.02602 | 0.669 | 7.9 | 5.3 | LRRC17 | leucine rich repeat containing 17 |
| 0.02602 | 0.757 | 70.1 | 53.0 | SNRNP70 | small nuclear ribonucleoprotein 70kDa (U1) |
| 0.02610 | 1.475 | 5.8 | 8.6 | TRPM2 | transient receptor potential cation channel, subfamily M, member 2 |
| 0.02618 | 1.170 | 12.2 | 14.3 | APOOL | apolipoprotein O-like |
| 0.02618 | 0.884 | 5.8 | 5.1 | NFXL1 | nuclear transcription factor, X-box binding-like 1 |
| 0.02622 | 0.793 | 10.5 | 8.3 | ZNF26 | zinc finger protein 26 |
| 0.02623 | 2.109 | 7.8 | 16.4 | LARP6 | La ribonucleoprotein domain family, member 6 |
| 0.02629 | 0.844 | 18.1 | 15.3 | ORMDL1 | ORM1-like 1 (S. cerevisiae) |
| 0.02630 | 0.602 | 9.8 | 5.9 | GAREML | GRB2 associated, regulator of MAPK1-like |
| 0.02633 | 1.382 | 9.9 | 13.7 | MGST3 | microsomal glutathione S-transferase 3 |
| 0.02635 | 0.779 | 11.7 | 9.1 | ZNF318 | zinc finger protein 318 |
| 0.02636 | 1.380 | 20.7 | 28.6 | ANKRD40 | ankyrin repeat domain 40 |
| 0.02637 | 3.215 | 7.3 | 23.5 | LOC102724850 | uncharacterized LOC102724850 |
| 0.02643 | 0.684 | 20.6 | 14.1 | LOC101927340 | uncharacterized LOC101927340 |
| 0.02643 | 0.715 | 9.4 | 6.7 | TDG | thymine-DNA glycosylase |
| 0.02644 | 0.783 | 6.7 | 5.3 | ZNF738 | zinc finger protein 738 |
| 0.02644 | 0.634 | 36.8 | 23.4 | IVNS1ABP | influenza virus NS1A binding protein |
| 0.02655 | 1.333 | 9.4 | 12.5 | MAPK9 | mitogen-activated protein kinase 9 |
| 0.02663 | 0.740 | 17.5 | 12.9 | PIK3R2 | phosphoinositide-3-kinase, regulatory subunit 2 (beta) |
| 0.02664 | 0.780 | 20.1 | 15.7 | SMARCB1 | SWI/SNF related, matrix associated, actin dependent regulator of chromatin, subfamily b, member 1 |
| 0.02665 | 1.241 | 5.5 | 6.8 | RNF217 | ring finger protein 217 |
| 0.02668 | 0.697 | 19.7 | 13.8 | DDX56 | DEAD (Asp-Glu-Ala-Asp) box helicase 56 |
| 0.02669 | 0.573 | 187.5 | 107.5 | LOC102725006 | uncharacterized LOC102725006 |
| 0.02670 | 1.717 | 10.4 | 17.8 | NCS1 | neuronal calcium sensor 1 |
| 0.02674 | 0.794 | 14.2 | 11.3 | CLK2 | CDC-like kinase 2 |
| 0.02676 | 1.481 | 13.2 | 19.6 | LMBRD1 | LMBR1 domain containing 1 |
| 0.02679 | 0.772 | 37.4 | 28.9 | RBM4 | RNA binding motif protein 4 |
| 0.02685 | 1.967 | 14.8 | 29.2 | TTLL7 | tubulin tyrosine ligase-like family, member 7 |
| 0.02687 | 1.679 | 12.4 | 20.8 | PCGF5 | polycomb group ring finger 5 |
| 0.02689 | 0.745 | 15.9 | 11.8 | ZNF84 | zinc finger protein 84 |
| 0.02702 | 1.339 | 9.3 | 12.4 | TMEM126B | transmembrane protein 126B |
| 0.02705 | 0.643 | 11.4 | 7.3 | ANGPT2 | angiopoietin 2 |
| 0.02706 | 1.312 | 62.0 | 81.3 | GABARAP | GABA(A) receptor-associated protein |
| 0.02711 | 0.843 | 30.1 | 25.3 | NXF1 | nuclear RNA export factor 1 |
| 0.02718 | 0.767 | 20.5 | 15.7 | FARP1 | FERM, RhoGEF (ARHGEF) and pleckstrin domain protein 1 (chondrocyte-derived) |
| 0.02727 | 0.690 | 25.6 | 17.7 | MYEF2 | myelin expression factor 2 |
| 0.02729 | 0.728 | 14.1 | 10.2 | RBM15 | RNA binding motif protein 15 |
| 0.02736 | 0.785 | 43.7 | 34.3 | SRSF1 | serine/arginine-rich splicing factor 1 |
| 0.02736 | 0.883 | 6.0 | 5.3 | PPAN-P2RY11 | PPAN-P2RY11 readthrough |
| 0.02738 | 1.357 | 5.3 | 7.3 | TARSL2 | threonyl-tRNA synthetase-like 2 |
| 0.02740 | 0.825 | 10.3 | 8.5 | TTI1 | TELO2 interacting protein 1 |
| 0.02742 | 0.769 | 29.8 | 22.9 | ZNF146 | zinc finger protein 146 |
| 0.02744 | 0.824 | 6.8 | 5.6 | LRRC75A | leucine rich repeat containing 75A |
| 0.02746 | 0.860 | 70.9 | 61.0 | RBM39 | RNA binding motif protein 39 |
| 0.02750 | 4.438 | 6.6 | 29.1 | CARNS1 | carnosine synthase 1 |
| 0.02752 | 1.237 | 5.4 | 6.7 | TRIQK | triple QxxK/R motif containing |
| 0.02755 | 0.758 | 11.2 | 8.5 | TSKU | tsukushi, small leucine rich proteoglycan |
| 0.02768 | 1.535 | 11.7 | 17.9 | ZNF385A | zinc finger protein 385A |
| 0.02780 | 0.661 | 14.1 | 9.3 | CASP3 | caspase 3, apoptosis-related cysteine peptidase |
| 0.02790 | 1.430 | 7.1 | 10.2 | DIP2C | DIP2 disco-interacting protein 2 homolog C (Drosophila) |
| 0.02793 | 0.675 | 8.4 | 5.7 | SHF | Src homology 2 domain containing F |
| 0.02799 | 1.385 | 28.8 | 39.9 | ATP6V1F | ATPase, H+ transporting, lysosomal 14kDa, V1 subunit F |
| 0.02802 | 0.873 | 5.9 | 5.1 | SDSL | serine dehydratase-like |
| 0.02803 | 0.609 | 58.7 | 35.8 | PHLDA1 | pleckstrin homology-like domain, family A, member 1 |
| 0.02805 | 1.601 | 21.2 | 33.9 | GRN | granulin |
| 0.02808 | 0.526 | 16.1 | 8.5 | MAGI1 | membrane associated guanylate kinase, WW and PDZ domain containing 1 |
| 0.02809 | 1.411 | 21.0 | 29.6 | MDH1 | malate dehydrogenase 1, NAD (soluble) |
| 0.02811 | 0.609 | 43.0 | 26.2 | ACAP3 | ArfGAP with coiled-coil, ankyrin repeat and PH domains 3 |
| 0.02814 | 1.252 | 33.3 | 41.7 | TGOLN2 | trans-golgi network protein 2 |
| 0.02815 | 1.729 | 7.4 | 12.8 | SLFN5 | schlafen family member 5 |
| 0.02817 | 1.450 | 5.9 | 8.6 | ARHGAP30 | Rho GTPase activating protein 30 |
| 0.02821 | 0.768 | 7.3 | 5.6 | ENTHD2 | ENTH domain containing 2 |
| 0.02822 | 1.289 | 6.2 | 8.0 | C19orf70 | chromosome 19 open reading frame 70 |
| 0.02825 | 2.463 | 507.5 | 1249.8 | CDR1 | cerebellar degeneration-related protein 1, 34kDa |
| 0.02827 | 0.802 | 17.9 | 14.4 | ZC3H14 | zinc finger CCCH-type containing 14 |
| 0.02827 | 1.810 | 15.4 | 27.8 | CDKN1A | cyclin-dependent kinase inhibitor 1A (p21, Cip1) |
| 0.02830 | 0.657 | 25.3 | 16.6 | NOP56 | NOP56 ribonucleoprotein |
| 0.02833 | 1.219 | 5.4 | 6.6 | SGPP1 | sphingosine-1-phosphate phosphatase 1 |
| 0.02838 | 1.606 | 8.2 | 13.1 | CYSTM1 | cysteine-rich transmembrane module containing 1 |
| 0.02840 | 0.852 | 14.7 | 12.5 | SMURF2 | SMAD specific E3 ubiquitin protein ligase 2 |
| 0.02841 | 0.811 | 6.9 | 5.6 | DHX34 | DEAH (Asp-Glu-Ala-His) box polypeptide 34 |
| 0.02844 | 0.685 | 18.9 | 12.9 | PMS2P9 | postmeiotic segregation increased 2 pseudogene 9 |
| 0.02847 | 0.732 | 25.7 | 18.8 | CLASP1 | cytoplasmic linker associated protein 1 |
| 0.02850 | 0.765 | 11.8 | 9.0 | OGG1 | 8-oxoguanine DNA glycosylase |
| 0.02856 | 2.200 | 5.4 | 11.8 | KLK6 | kallikrein-related peptidase 6 |
| 0.02857 | 4.257 | 6.1 | 25.8 | MAG | myelin associated glycoprotein |
| 0.02859 | 2.165 | 5.0 | 10.8 | SH3GL2 | SH3-domain GRB2-like 2 |
| 0.02864 | 0.768 | 6.6 | 5.1 | ACHE | acetylcholinesterase (Yt blood group) |
| 0.02870 | 1.637 | 31.0 | 50.8 | CAMK2N1 | calcium/calmodulin-dependent protein kinase II inhibitor 1 |
| 0.02871 | 1.693 | 7.5 | 12.7 | FCGR3B | Fc fragment of IgG, low affinity IIIb, receptor (CD16b) |
| 0.02876 | 0.728 | 8.0 | 5.8 | TEX10 | testis expressed 10 |
| 0.02877 | 0.847 | 6.3 | 5.3 | LOC102723342 | uncharacterized LOC102723342 |
| 0.02877 | 1.632 | 24.2 | 39.5 | GAS7 | growth arrest-specific 7 |
| 0.02881 | 0.783 | 23.7 | 18.5 | PHC1 | polyhomeotic homolog 1 (Drosophila) |
| 0.02885 | 0.693 | 7.9 | 5.5 | ZNF781 | zinc finger protein 781 |
| 0.02890 | 1.625 | 11.7 | 19.0 | MVB12B | multivesicular body subunit 12B |
| 0.02897 | 1.268 | 13.2 | 16.8 | UBA3 | ubiquitin-like modifier activating enzyme 3 |
| 0.02903 | 0.667 | 14.1 | 9.4 | SUZ12P1 | suppressor of zeste 12 homolog pseudogene 1 |
| 0.02903 | 1.647 | 60.7 | 100.0 | HLA-E | major histocompatibility complex, class I, E |
| 0.02904 | 1.413 | 7.5 | 10.7 | HABP4 | hyaluronan binding protein 4 |
| 0.02904 | 0.880 | 5.8 | 5.1 | ZNF786 | zinc finger protein 786 |
| 0.02914 | 1.254 | 5.7 | 7.1 | ICT1 | immature colon carcinoma transcript 1 |
| 0.02915 | 3.298 | 8.6 | 28.4 | CDK18 | cyclin-dependent kinase 18 |
| 0.02919 | 1.402 | 30.8 | 43.2 | REEP5 | receptor accessory protein 5 |
| 0.02919 | 0.692 | 22.2 | 15.4 | LOC102725128 | speedy protein E2-like |
| 0.02922 | 0.799 | 20.6 | 16.4 | RCOR3 | REST corepressor 3 |
| 0.02924 | 1.394 | 6.2 | 8.6 | PLEK | pleckstrin |
| 0.02925 | 2.310 | 23.3 | 53.8 | APLP1 | amyloid beta (A4) precursor-like protein 1 |
| 0.02925 | 1.421 | 9.1 | 13.0 | DNASE2 | deoxyribonuclease II, lysosomal |
| 0.02928 | 1.556 | 5.1 | 8.0 | PRRG1 | proline rich Gla (G-carboxyglutamic acid) 1 |
| 0.02930 | 1.284 | 6.4 | 8.2 | NEU1 | sialidase 1 (lysosomal sialidase) |
| 0.02930 | 0.795 | 45.0 | 35.8 | ACIN1 | apoptotic chromatin condensation inducer 1 |
| 0.02932 | 1.935 | 18.3 | 35.3 | IFI44 | interferon-induced protein 44 |
| 0.02934 | 0.704 | 11.1 | 7.8 | LBH | limb bud and heart development |
| 0.02938 | 1.882 | 5.3 | 10.0 | ST18 | suppression of tumorigenicity 18, zinc finger |
| 0.02938 | 1.151 | 11.9 | 13.7 | RAB3GAP1 | RAB3 GTPase activating protein subunit 1 (catalytic) |
| 0.02939 | 0.830 | 7.4 | 6.1 | SRRD | SRR1 domain containing |
| 0.02943 | 1.987 | 18.8 | 37.3 | MS4A7 | membrane-spanning 4-domains, subfamily A, member 7 |
| 0.02947 | 0.733 | 15.6 | 11.4 | GJC1 | gap junction protein, gamma 1, 45kDa |
| 0.02947 | 1.969 | 34.5 | 67.9 | FCGR3A | Fc fragment of IgG, low affinity IIIa, receptor (CD16a) |
| 0.02949 | 1.728 | 5.0 | 8.7 | PPP1R16B | protein phosphatase 1, regulatory subunit 16B |
| 0.02950 | 1.156 | 5.1 | 5.9 | HS1BP3 | HCLS1 binding protein 3 |
| 0.02954 | 1.723 | 28.9 | 49.8 | ITPKB | inositol-trisphosphate 3-kinase B |
| 0.02954 | 1.464 | 5.0 | 7.3 | TUBA4A | tubulin, alpha 4a |
| 0.02957 | 0.811 | 19.9 | 16.1 | PGAM5 | phosphoglycerate mutase family member 5 |
| 0.02960 | 1.286 | 7.3 | 9.4 | PPIP5K1 | diphosphoinositol pentakisphosphate kinase 1 |
| 0.02968 | 0.738 | 31.3 | 23.1 | FLJ31306 | uncharacterized LOC379025 |
| 0.02972 | 1.386 | 9.5 | 13.1 | RRAGC | Ras-related GTP binding C |
| 0.02974 | 0.728 | 41.1 | 29.9 | SPDYE6 | speedy/RINGO cell cycle regulator family member E6 |
| 0.02981 | 1.319 | 15.7 | 20.8 | TRAK2 | trafficking protein, kinesin binding 2 |
| 0.02989 | 0.819 | 10.0 | 8.2 | MOB4 | MOB family member 4, phocein |
| 0.02992 | 0.728 | 10.3 | 7.5 | UGGT2 | UDP-glucose glycoprotein glucosyltransferase 2 |
| 0.02995 | 0.776 | 30.7 | 23.8 | DHX9 | DEAH (Asp-Glu-Ala-His) box helicase 9 |
| 0.02998 | 1.315 | 22.4 | 29.4 | DCTN1 | dynactin 1 |
| 0.03000 | 3.201 | 5.7 | 18.2 | SYN2 | synapsin II |
| 0.03004 | 1.558 | 36.3 | 56.5 | PRNP | prion protein |
| 0.03008 | 1.177 | 16.8 | 19.8 | NUDC | nudC nuclear distribution protein |
| 0.03012 | 0.766 | 16.0 | 12.3 | CRYBB2P1 | crystallin, beta B2 pseudogene 1 |
| 0.03015 | 5.161 | 112.5 | 580.4 | MBP | myelin basic protein |
| 0.03030 | 0.860 | 14.9 | 12.8 | PCM1 | pericentriolar material 1 |
| 0.03043 | 0.754 | 17.9 | 13.5 | ZNF276 | zinc finger protein 276 |
| 0.03045 | 0.817 | 29.9 | 24.4 | SLC35E2B | solute carrier family 35, member E2B |
| 0.03046 | 0.744 | 8.4 | 6.3 | RNF2 | ring finger protein 2 |
| 0.03048 | 1.516 | 21.5 | 32.6 | LAP3 | leucine aminopeptidase 3 |
| 0.03054 | 0.783 | 6.9 | 5.4 | ZFP82 | ZFP82 zinc finger protein |
| 0.03054 | 1.260 | 40.1 | 50.5 | ATP6V0C | ATPase, H+ transporting, lysosomal 16kDa, V0 subunit c |
| 0.03056 | 0.825 | 6.6 | 5.4 | RAD9A | RAD9 homolog A (S. pombe) |
| 0.03062 | 1.472 | 6.3 | 9.3 | MAFK | v-maf avian musculoaponeurotic fibrosarcoma oncogene homolog K |
| 0.03064 | 0.807 | 6.9 | 5.6 | ZNF14 | zinc finger protein 14 |
| 0.03070 | 0.884 | 42.7 | 37.8 | EWSR1 | EWS RNA-binding protein 1 |
| 0.03073 | 0.832 | 12.6 | 10.5 | MRPL30 | mitochondrial ribosomal protein L30 |
| 0.03079 | 0.700 | 19.7 | 13.8 | LOC101930277 | putative speedy protein-like protein LOC442572-like |
| 0.03081 | 1.271 | 8.9 | 11.3 | CLIP4 | CAP-GLY domain containing linker protein family, member 4 |
| 0.03095 | 0.772 | 12.1 | 9.3 | CROCCP3 | ciliary rootlet coiled-coil, rootletin pseudogene 3 |
| 0.03103 | 0.819 | 44.5 | 36.5 | FNBP4 | formin binding protein 4 |
| 0.03105 | 0.617 | 10.7 | 6.6 | NOL4 | nucleolar protein 4 |
| 0.03107 | 0.678 | 34.6 | 23.4 | PHF14 | PHD finger protein 14 |
| 0.03113 | 0.745 | 49.5 | 36.9 | SPDYE2 | speedy/RINGO cell cycle regulator family member E2 |
| 0.03122 | 0.782 | 52.6 | 41.1 | RBM6 | RNA binding motif protein 6 |
| 0.03125 | 1.316 | 5.3 | 7.0 | SPNS2 | spinster homolog 2 (Drosophila) |
| 0.03134 | 1.226 | 21.6 | 26.5 | CCSER2 | coiled-coil serine-rich protein 2 |
| 0.03135 | 0.701 | 31.2 | 21.9 | PAXBP1 | PAX3 and PAX7 binding protein 1 |
| 0.03137 | 0.583 | 31.5 | 18.4 | ZNF462 | zinc finger protein 462 |
| 0.03141 | 0.803 | 56.4 | 45.3 | ATN1 | atrophin 1 |
| 0.03143 | 0.777 | 35.1 | 27.3 | TRA2A | transformer 2 alpha homolog (Drosophila) |
| 0.03144 | 1.361 | 5.0 | 6.8 | CPEB1 | cytoplasmic polyadenylation element binding protein 1 |
| 0.03154 | 2.362 | 11.5 | 27.2 | KCNMB4 | potassium large conductance calcium-activated channel, subfamily M, beta member 4 |
| 0.03154 | 0.759 | 8.5 | 6.5 | VCPKMT | valosin containing protein lysine (K) methyltransferase |
| 0.03160 | 1.266 | 52.8 | 66.8 | DAZAP2 | DAZ associated protein 2 |
| 0.03161 | 3.046 | 9.9 | 30.0 | EFHD1 | EF-hand domain family, member D1 |
| 0.03167 | 1.324 | 34.5 | 45.7 | BNIP3L | BCL2/adenovirus E1B 19kDa interacting protein 3-like |
| 0.03167 | 1.653 | 5.0 | 8.3 | RAB11FIP4 | RAB11 family interacting protein 4 (class II) |
| 0.03168 | 0.715 | 38.6 | 27.6 | CHD9 | chromodomain helicase DNA binding protein 9 |
| 0.03177 | 2.537 | 33.0 | 83.7 | CLDND1 | claudin domain containing 1 |
| 0.03179 | 1.319 | 6.5 | 8.6 | C9orf91 | chromosome 9 open reading frame 91 |
| 0.03182 | 1.254 | 77.3 | 96.9 | OAZ1 | ornithine decarboxylase antizyme 1 |
| 0.03184 | 1.508 | 14.2 | 21.4 | WIPF1 | WAS/WASL interacting protein family, member 1 |
| 0.03188 | 0.801 | 6.7 | 5.3 | PDSS2 | prenyl (decaprenyl) diphosphate synthase, subunit 2 |
| 0.03189 | 1.379 | 12.7 | 17.5 | CREBL2 | cAMP responsive element binding protein-like 2 |
| 0.03200 | 0.444 | 23.0 | 10.2 | SEZ6L | seizure related 6 homolog (mouse)-like |
| 0.03200 | 0.708 | 9.1 | 6.5 | ZNF678 | zinc finger protein 678 |
| 0.03205 | 1.389 | 5.4 | 7.6 | HELZ2 | helicase with zinc finger 2, transcriptional coactivator |
| 0.03206 | 1.453 | 12.5 | 18.2 | MAF | v-maf avian musculoaponeurotic fibrosarcoma oncogene homolog |
| 0.03211 | 1.408 | 23.5 | 33.1 | BRI3 | brain protein I3 |
| 0.03220 | 1.410 | 15.8 | 22.2 | MAPRE3 | microtubule-associated protein, RP/EB family, member 3 |
| 0.03221 | 0.759 | 146.1 | 110.9 | HNRNPA1 | heterogeneous nuclear ribonucleoprotein A1 |
| 0.03225 | 0.774 | 73.9 | 57.2 | RBMX | RNA binding motif protein, X-linked |
| 0.03225 | 2.170 | 12.2 | 26.5 | LY6E | lymphocyte antigen 6 complex, locus E |
| 0.03227 | 1.591 | 18.8 | 29.9 | RHOU | ras homolog family member U |
| 0.03228 | 1.591 | 15.5 | 24.6 | BNIP3 | BCL2/adenovirus E1B 19kDa interacting protein 3 |
| 0.03230 | 0.726 | 7.5 | 5.4 | RNF152 | ring finger protein 152 |
| 0.03234 | 0.549 | 29.2 | 16.0 | SNORA11D | small nucleolar RNA, H/ACA box 11D |
| 0.03237 | 1.331 | 11.7 | 15.5 | TNIP1 | TNFAIP3 interacting protein 1 |
| 0.03240 | 1.261 | 40.1 | 50.5 | SKP1 | S-phase kinase-associated protein 1 |
| 0.03241 | 1.246 | 10.0 | 12.5 | SLC35A5 | solute carrier family 35, member A5 |
| 0.03243 | 0.749 | 21.1 | 15.8 | CHD3 | chromodomain helicase DNA binding protein 3 |
| 0.03246 | 1.330 | 6.0 | 8.0 | MOB3C | MOB kinase activator 3C |
| 0.03258 | 0.686 | 31.1 | 21.3 | HNRNPU-AS1 | HNRNPU antisense RNA 1 |
| 0.03262 | 0.840 | 36.1 | 30.4 | VASH1 | vasohibin 1 |
| 0.03268 | 1.241 | 23.2 | 28.8 | MAP7D1 | MAP7 domain containing 1 |
| 0.03273 | 0.800 | 21.3 | 17.1 | STRN4 | striatin, calmodulin binding protein 4 |
| 0.03287 | 0.784 | 28.6 | 22.4 | UBA2 | ubiquitin-like modifier activating enzyme 2 |
| 0.03292 | 1.872 | 7.4 | 13.8 | FBXL16 | F-box and leucine-rich repeat protein 16 |
| 0.03300 | 1.384 | 6.3 | 8.7 | FBXW4 | F-box and WD repeat domain containing 4 |
| 0.03301 | 0.650 | 24.8 | 16.2 | LOC101929491 | uncharacterized LOC101929491 |
| 0.03301 | 0.552 | 77.5 | 42.8 | NOVA1 | neuro-oncological ventral antigen 1 |
| 0.03301 | 0.733 | 21.6 | 15.8 | ANAPC7 | anaphase promoting complex subunit 7 |
| 0.03301 | 0.856 | 6.3 | 5.4 | PLAGL2 | pleiomorphic adenoma gene-like 2 |
| 0.03307 | 0.792 | 8.7 | 6.9 | ZNF275 | zinc finger protein 275 |
| 0.03323 | 1.511 | 13.2 | 19.9 | RNF220 | ring finger protein 220 |
| 0.03327 | 0.819 | 10.9 | 8.9 | PRR12 | proline rich 12 |
| 0.03332 | 1.664 | 5.5 | 9.1 | FPR3 | formyl peptide receptor 3 |
| 0.03332 | 0.807 | 6.5 | 5.3 | NRARP | NOTCH-regulated ankyrin repeat protein |
| 0.03335 | 0.851 | 19.9 | 17.0 | CREBBP | CREB binding protein |
| 0.03337 | 0.440 | 51.4 | 22.6 | GRIA2 | glutamate receptor, ionotropic, AMPA 2 |
| 0.03339 | 0.528 | 19.5 | 10.3 | TRAF4 | TNF receptor-associated factor 4 |
| 0.03340 | 0.817 | 8.0 | 6.5 | ZBED4 | zinc finger, BED-type containing 4 |
| 0.03344 | 4.531 | 6.3 | 28.5 | SLC17A7 | solute carrier family 17 (vesicular glutamate transporter), member 7 |
| 0.03347 | 1.484 | 9.3 | 13.9 | SPTLC2 | serine palmitoyltransferase, long chain base subunit 2 |
| 0.03349 | 0.677 | 8.4 | 5.7 | AC019181.2 | uncharacterized LOC101929633 |
| 0.03355 | 1.343 | 8.9 | 12.0 | SLK | STE20-like kinase |
| 0.03361 | 0.790 | 18.9 | 14.9 | MAGI2 | membrane associated guanylate kinase, WW and PDZ domain containing 2 |
| 0.03364 | 1.844 | 6.1 | 11.3 | AMPD3 | adenosine monophosphate deaminase 3 |
| 0.03367 | 1.440 | 5.3 | 7.6 | HSD17B3 | hydroxysteroid (17-beta) dehydrogenase 3 |
| 0.03368 | 0.724 | 14.2 | 10.3 | ZNF626 | zinc finger protein 626 |
| 0.03370 | 0.763 | 6.7 | 5.1 | LOC101927260 | uncharacterized LOC101927260 |
| 0.03375 | 1.377 | 5.0 | 6.9 | RASGRF2 | Ras protein-specific guanine nucleotide-releasing factor 2 |
| 0.03376 | 0.728 | 10.0 | 7.3 | ZNF749 | zinc finger protein 749 |
| 0.03381 | 0.857 | 6.0 | 5.1 | PDF | peptide deformylase (mitochondrial) |
| 0.03384 | 0.784 | 6.5 | 5.1 | C6orf147 | chromosome 6 open reading frame 147 |
| 0.03384 | 1.218 | 6.2 | 7.5 | C5orf22 | chromosome 5 open reading frame 22 |
| 0.03384 | 0.807 | 29.0 | 23.4 | SRR | serine racemase |
| 0.03386 | 1.277 | 17.1 | 21.8 | LAMTOR1 | late endosomal/lysosomal adaptor, MAPK and MTOR activator 1 |
| 0.03398 | 0.558 | 19.9 | 11.1 | DPF3 | D4, zinc and double PHD fingers, family 3 |
| 0.03399 | 0.775 | 22.0 | 17.1 | PA2G4 | proliferation-associated 2G4, 38kDa |
| 0.03401 | 0.720 | 13.0 | 9.3 | ATP6V1G2-DDX39B | ATP6V1G2-DDX39B readthrough (NMD candidate) |
| 0.03404 | 1.203 | 9.6 | 11.5 | NAPG | N-ethylmaleimide-sensitive factor attachment protein, gamma |
| 0.03407 | 2.646 | 29.6 | 78.3 | MTURN | maturin, neural progenitor differentiation regulator homolog (Xenopus) |
| 0.03411 | 1.410 | 5.6 | 7.9 | LINC00639 | long intergenic non-protein coding RNA 639 |
| 0.03414 | 1.532 | 8.8 | 13.5 | IFI27L2 | interferon, alpha-inducible protein 27-like 2 |
| 0.03418 | 0.821 | 6.7 | 5.5 | CYP2R1 | cytochrome P450, family 2, subfamily R, polypeptide 1 |
| 0.03422 | 0.808 | 8.1 | 6.5 | SARM1 | sterile alpha and TIR motif containing 1 |
| 0.03427 | 1.493 | 6.4 | 9.6 | CBX7 | chromobox homolog 7 |
| 0.03432 | 0.803 | 6.4 | 5.1 | ZNRF1 | zinc and ring finger 1, E3 ubiquitin protein ligase |
| 0.03434 | 1.681 | 6.6 | 11.1 | FCGR1B | Fc fragment of IgG, high affinity Ib, receptor (CD64) |
| 0.03435 | 0.686 | 8.6 | 5.9 | C19orf26 | chromosome 19 open reading frame 26 |
| 0.03443 | 0.792 | 29.0 | 23.0 | NME4 | NME/NM23 nucleoside diphosphate kinase 4 |
| 0.03453 | 1.667 | 10.6 | 17.7 | EPB41L1 | erythrocyte membrane protein band 4.1-like 1 |
| 0.03453 | 1.443 | 42.5 | 61.3 | SORT1 | sortilin 1 |
| 0.03454 | 1.219 | 20.6 | 25.2 | RTFDC1 | replication termination factor 2 domain containing 1 |
| 0.03463 | 0.654 | 14.2 | 9.3 | KIAA0895L | KIAA0895-like |
| 0.03468 | 0.818 | 43.9 | 35.9 | HNRNPUL1 | heterogeneous nuclear ribonucleoprotein U-like 1 |
| 0.03469 | 2.241 | 15.6 | 34.9 | RTN1 | reticulon 1 |
| 0.03470 | 1.432 | 131.2 | 187.8 | GLUL | glutamate-ammonia ligase |
| 0.03483 | 0.723 | 15.6 | 11.3 | TRO | trophinin |
| 0.03483 | 1.206 | 12.3 | 14.8 | TRAPPC10 | trafficking protein particle complex 10 |
| 0.03489 | 0.730 | 19.6 | 14.3 | ERV3-1 | endogenous retrovirus group 3, member 1 |
| 0.03492 | 1.387 | 5.0 | 6.9 | PLD4 | phospholipase D family, member 4 |
| 0.03503 | 1.869 | 14.1 | 26.3 | ITGB2 | integrin, beta 2 (complement component 3 receptor 3 and 4 subunit) |
| 0.03506 | 0.639 | 54.8 | 35.0 | ILF3 | interleukin enhancer binding factor 3, 90kDa |
| 0.03508 | 1.450 | 6.2 | 8.9 | TAPSAR1 | TAP1 and PSMB8 antisense RNA 1 |
| 0.03511 | 0.750 | 20.2 | 15.2 | ZNF117 | zinc finger protein 117 |
| 0.03519 | 1.563 | 6.7 | 10.4 | PSMB9 | proteasome (prosome, macropain) subunit, beta type, 9 |
| 0.03526 | 1.261 | 15.1 | 19.0 | EMC4 | ER membrane protein complex subunit 4 |
| 0.03526 | 1.596 | 5.2 | 8.4 | CLCN4 | chloride channel, voltage-sensitive 4 |
| 0.03527 | 1.695 | 9.3 | 15.7 | LGALS9 | lectin, galactoside-binding, soluble, 9 |
| 0.03535 | 1.361 | 18.0 | 24.5 | CNPY3 | canopy FGF signaling regulator 3 |
| 0.03537 | 4.288 | 11.2 | 47.9 | NRGN | neurogranin (protein kinase C substrate, RC3) |
| 0.03537 | 4.016 | 9.9 | 40.0 | CLDN11 | claudin 11 |
| 0.03542 | 0.682 | 7.9 | 5.4 | NAV2-AS4 | NAV2 antisense RNA 4 |
| 0.03554 | 2.511 | 5.6 | 14.1 | LOC101929249 | uncharacterized LOC101929249 |
| 0.03555 | 0.711 | 7.2 | 5.2 | CAMK2N2 | calcium/calmodulin-dependent protein kinase II inhibitor 2 |
| 0.03561 | 0.805 | 53.8 | 43.3 | RBM5 | RNA binding motif protein 5 |
| 0.03562 | 1.653 | 32.0 | 52.8 | ABCA1 | ATP-binding cassette, sub-family A (ABC1), member 1 |
| 0.03565 | 1.302 | 12.3 | 16.0 | ZNFX1 | zinc finger, NFX1-type containing 1 |
| 0.03578 | 0.653 | 8.4 | 5.5 | BLM | Bloom syndrome, RecQ helicase-like |
| 0.03580 | 3.829 | 12.8 | 48.9 | MOBP | myelin-associated oligodendrocyte basic protein |
| 0.03581 | 0.670 | 23.9 | 16.0 | STK17A | serine/threonine kinase 17a |
| 0.03587 | 0.834 | 6.2 | 5.2 | PUSL1 | pseudouridylate synthase-like 1 |
| 0.03591 | 0.848 | 6.2 | 5.3 | LNX2 | ligand of numb-protein X 2 |
| 0.03592 | 1.964 | 11.0 | 21.6 | CTSH | cathepsin H |
| 0.03600 | 0.687 | 20.4 | 14.0 | SYNE2 | spectrin repeat containing, nuclear envelope 2 |
| 0.03614 | 0.816 | 7.3 | 6.0 | RBMX2 | RNA binding motif protein, X-linked 2 |
| 0.03620 | 1.261 | 49.8 | 62.8 | IL6ST | interleukin 6 signal transducer (gp130, oncostatin M receptor) |
| 0.03625 | 0.715 | 9.8 | 7.0 | ZNF736 | zinc finger protein 736 |
| 0.03626 | 1.354 | 9.6 | 12.9 | CYLD | cylindromatosis (turban tumor syndrome) |
| 0.03626 | 0.686 | 19.3 | 13.2 | ZNF226 | zinc finger protein 226 |
| 0.03640 | 0.699 | 10.3 | 7.2 | INTS7 | integrator complex subunit 7 |
| 0.03640 | 0.840 | 12.3 | 10.3 | KMT2B | lysine (K)-specific methyltransferase 2B |
| 0.03642 | 0.697 | 13.5 | 9.4 | TCF3 | transcription factor 3 |
| 0.03644 | 1.291 | 5.0 | 6.5 | LOC101929513 | uncharacterized LOC101929513 |
| 0.03645 | 0.620 | 12.6 | 7.8 | ST7-AS2 | ST7 antisense RNA 2 |
| 0.03647 | 1.938 | 5.1 | 9.8 | PHYHIP | phytanoyl-CoA 2-hydroxylase interacting protein |
| 0.03647 | 1.261 | 8.1 | 10.2 | SLC39A11 | solute carrier family 39, member 11 |
| 0.03648 | 1.574 | 6.5 | 10.3 | NXPE3 | neurexophilin and PC-esterase domain family, member 3 |
| 0.03654 | 2.333 | 5.2 | 12.2 | AK5 | adenylate kinase 5 |
| 0.03658 | 0.789 | 8.8 | 6.9 | FAM136A | family with sequence similarity 136, member A |
| 0.03659 | 0.779 | 63.3 | 49.3 | SRSF11 | serine/arginine-rich splicing factor 11 |
| 0.03659 | 1.310 | 5.0 | 6.6 | MIR635 | microRNA 635 |
| 0.03664 | 1.318 | 5.0 | 6.6 | CHADL | chondroadherin-like |
| 0.03667 | 2.753 | 19.5 | 53.7 | HLA-DQA1 | major histocompatibility complex, class II, DQ alpha 1 |
| 0.03670 | 0.776 | 6.5 | 5.0 | LOC102724641 | glycine, alanine and asparagine-rich protein-like |
| 0.03679 | 1.232 | 6.5 | 8.0 | SLC25A16 | solute carrier family 25 (mitochondrial carrier; Graves disease autoantigen), member 16 |
| 0.03679 | 0.833 | 7.0 | 5.8 | ZBTB2 | zinc finger and BTB domain containing 2 |
| 0.03687 | 1.295 | 23.3 | 30.2 | EIF2AK2 | eukaryotic translation initiation factor 2-alpha kinase 2 |
| 0.03688 | 0.700 | 7.9 | 5.5 | POLE | polymerase (DNA directed), epsilon, catalytic subunit |
| 0.03692 | 1.758 | 6.3 | 11.1 | SLCO3A1 | solute carrier organic anion transporter family, member 3A1 |
| 0.03698 | 0.820 | 35.6 | 29.2 | CDC42SE1 | CDC42 small effector 1 |
| 0.03698 | 1.776 | 5.2 | 9.3 | SLC7A14 | solute carrier family 7, member 14 |
| 0.03698 | 0.521 | 96.2 | 50.1 | NES | nestin |
| 0.03700 | 1.446 | 8.1 | 11.7 | SNX9 | sorting nexin 9 |
| 0.03709 | 1.744 | 165.0 | 287.8 | HLA-B | major histocompatibility complex, class I, B |
| 0.03711 | 1.130 | 5.2 | 5.9 | PIGN | phosphatidylinositol glycan anchor biosynthesis, class N |
| 0.03713 | 1.894 | 6.0 | 11.5 | ODF3B | outer dense fiber of sperm tails 3B |
| 0.03719 | 1.881 | 9.9 | 18.6 | ATP8A1 | ATPase, aminophospholipid transporter (APLT), class I, type 8A, member 1 |
| 0.03723 | 1.354 | 6.9 | 9.4 | NUDCD2 | NudC domain containing 2 |
| 0.03724 | 0.749 | 12.8 | 9.6 | DDX55 | DEAD (Asp-Glu-Ala-Asp) box polypeptide 55 |
| 0.03728 | 1.235 | 5.3 | 6.6 | IRF8 | interferon regulatory factor 8 |
| 0.03729 | 0.649 | 17.0 | 11.0 | CACNG7 | calcium channel, voltage-dependent, gamma subunit 7 |
| 0.03731 | 1.259 | 7.4 | 9.3 | FUT11 | fucosyltransferase 11 (alpha (1,3) fucosyltransferase) |
| 0.03733 | 0.616 | 16.3 | 10.0 | MPPED2 | metallophosphoesterase domain containing 2 |
| 0.03733 | 0.794 | 9.8 | 7.8 | LARP1B | La ribonucleoprotein domain family, member 1B |
| 0.03737 | 1.659 | 14.1 | 23.4 | ADAP2 | ArfGAP with dual PH domains 2 |
| 0.03741 | 0.828 | 17.5 | 14.5 | CCDC93 | coiled-coil domain containing 93 |
| 0.03742 | 1.238 | 20.9 | 25.9 | NBR1 | neighbor of BRCA1 gene 1 |
| 0.03745 | 0.496 | 21.5 | 10.7 | CACNG4 | calcium channel, voltage-dependent, gamma subunit 4 |
| 0.03747 | 0.708 | 25.2 | 17.9 | PCMTD2 | protein-L-isoaspartate (D-aspartate) O-methyltransferase domain containing 2 |
| 0.03749 | 1.218 | 5.9 | 7.2 | FAM21C | family with sequence similarity 21, member C |
| 0.03753 | 1.328 | 20.4 | 27.1 | FBXO7 | F-box protein 7 |
| 0.03755 | 1.934 | 14.1 | 27.2 | ANLN | anillin, actin binding protein |
| 0.03757 | 0.690 | 7.9 | 5.5 | AADAT | aminoadipate aminotransferase |
| 0.03763 | 0.835 | 6.3 | 5.3 | CDAN1 | codanin 1 |
| 0.03764 | 0.744 | 35.5 | 26.4 | PMS2P5 | postmeiotic segregation increased 2 pseudogene 5 |
| 0.03769 | 1.623 | 7.4 | 12.1 | FAM171A1 | family with sequence similarity 171, member A1 |
| 0.03771 | 0.811 | 6.4 | 5.2 | LOC102725017 | uncharacterized LOC102725017 |
| 0.03775 | 0.721 | 14.9 | 10.8 | ZNF428 | zinc finger protein 428 |
| 0.03778 | 0.804 | 19.9 | 16.0 | BCL2L2-PABPN1 | BCL2L2-PABPN1 readthrough |
| 0.03782 | 2.125 | 5.3 | 11.3 | RSAD2 | radical S-adenosyl methionine domain containing 2 |
| 0.03787 | 1.698 | 24.8 | 42.1 | RNASET2 | ribonuclease T2 |
| 0.03794 | 1.331 | 5.3 | 7.0 | SMIM4 | small integral membrane protein 4 |
| 0.03797 | 2.943 | 5.9 | 17.5 | PCSK6 | proprotein convertase subtilisin/kexin type 6 |
| 0.03801 | 1.344 | 26.1 | 35.1 | TMEM123 | transmembrane protein 123 |
| 0.03815 | 1.647 | 25.3 | 41.7 | PARP14 | poly (ADP-ribose) polymerase family, member 14 |
| 0.03817 | 1.431 | 9.5 | 13.6 | SELK | selenoprotein K |
| 0.03817 | 2.971 | 5.8 | 17.1 | SNCB | synuclein, beta |
| 0.03826 | 0.762 | 8.9 | 6.8 | GSS | glutathione synthetase |
| 0.03831 | 1.207 | 6.7 | 8.1 | MNT | MAX network transcriptional repressor |
| 0.03835 | 1.298 | 5.0 | 6.5 | CALY | calcyon neuron-specific vesicular protein |
| 0.03838 | 1.208 | 20.9 | 25.3 | SEL1L | sel-1 suppressor of lin-12-like (C. elegans) |
| 0.03846 | 1.194 | 5.4 | 6.5 | SETDB2 | SET domain, bifurcated 2 |
| 0.03851 | 1.473 | 7.8 | 11.6 | UBA7 | ubiquitin-like modifier activating enzyme 7 |
| 0.03859 | 0.777 | 18.9 | 14.7 | METTL3 | methyltransferase like 3 |
| 0.03864 | 1.313 | 5.4 | 7.1 | CCDC23 | coiled-coil domain containing 23 |
| 0.03876 | 0.555 | 12.4 | 6.9 | CNTFR | ciliary neurotrophic factor receptor |
| 0.03877 | 0.756 | 7.8 | 5.9 | FAM117B | family with sequence similarity 117, member B |
| 0.03882 | 0.841 | 33.2 | 27.9 | UBE2G2 | ubiquitin-conjugating enzyme E2G 2 |
| 0.03884 | 1.424 | 5.0 | 7.1 | TBC1D2 | TBC1 domain family, member 2 |
| 0.03888 | 1.543 | 24.1 | 37.2 | PLA2G16 | phospholipase A2, group XVI |
| 0.03889 | 1.687 | 5.6 | 9.5 | CPOX | coproporphyrinogen oxidase |
| 0.03892 | 1.159 | 6.4 | 7.5 | DOPEY2 | dopey family member 2 |
| 0.03894 | 0.817 | 52.0 | 42.4 | ZNF532 | zinc finger protein 532 |
| 0.03899 | 0.779 | 17.9 | 13.9 | PGBD2 | piggyBac transposable element derived 2 |
| 0.03899 | 0.655 | 8.3 | 5.4 | SAPCD2 | suppressor APC domain containing 2 |
| 0.03906 | 0.514 | 14.9 | 7.7 | SNORD46 | small nucleolar RNA, C/D box 46 |
| 0.03920 | 1.557 | 26.9 | 41.9 | SORL1 | sortilin-related receptor, L(DLR class) A repeats containing |
| 0.03921 | 0.784 | 6.6 | 5.2 | PRH2 | proline-rich protein HaeIII subfamily 2 |
| 0.03921 | 1.248 | 5.0 | 6.2 | ARHGDIG | Rho GDP dissociation inhibitor (GDI) gamma |
| 0.03922 | 0.815 | 27.6 | 22.5 | FBXO11 | F-box protein 11 |
| 0.03924 | 1.349 | 5.0 | 6.7 | LGI1 | leucine-rich, glioma inactivated 1 |
| 0.03927 | 1.399 | 15.2 | 21.3 | DNAJB2 | DnaJ (Hsp40) homolog, subfamily B, member 2 |
| 0.03927 | 1.406 | 5.0 | 7.1 | WBSCR17 | Williams-Beuren syndrome chromosome region 17 |
| 0.03928 | 0.871 | 29.5 | 25.7 | PDS5A | PDS5, regulator of cohesion maintenance, homolog A (S. cerevisiae) |
| 0.03934 | 1.263 | 5.2 | 6.5 | SQRDL | sulfide quinone reductase-like (yeast) |
| 0.03938 | 0.767 | 11.3 | 8.7 | CENPT | centromere protein T |
| 0.03940 | 0.747 | 7.7 | 5.8 | KIAA0408 | KIAA0408 |
| 0.03944 | 0.739 | 32.3 | 23.9 | ZNF493 | zinc finger protein 493 |
| 0.03947 | 1.336 | 7.3 | 9.7 | RAP2B | RAP2B, member of RAS oncogene family |
| 0.03948 | 1.178 | 5.0 | 5.9 | QPRT | quinolinate phosphoribosyltransferase |
| 0.03951 | 1.198 | 7.1 | 8.5 | ATG5 | autophagy related 5 |
| 0.03951 | 1.378 | 87.1 | 120.0 | SNORA74B | small nucleolar RNA, H/ACA box 74B |
| 0.03954 | 1.231 | 5.3 | 6.5 | TRIM69 | tripartite motif containing 69 |
| 0.03957 | 1.217 | 5.0 | 6.1 | EIF4E3 | eukaryotic translation initiation factor 4E family member 3 |
| 0.03960 | 3.467 | 9.6 | 33.4 | MX1 | myxovirus (influenza virus) resistance 1, interferon-inducible protein p78 (mouse) |
| 0.03966 | 1.232 | 5.1 | 6.3 | DBP | D site of albumin promoter (albumin D-box) binding protein |
| 0.03969 | 0.712 | 7.4 | 5.3 | ZNF93 | zinc finger protein 93 |
| 0.03971 | 2.035 | 5.1 | 10.3 | SERPINI1 | serpin peptidase inhibitor, clade I (neuroserpin), member 1 |
| 0.03979 | 0.818 | 19.3 | 15.8 | ZMYM2 | zinc finger, MYM-type 2 |
| 0.03980 | 1.548 | 6.3 | 9.8 | PIK3AP1 | phosphoinositide-3-kinase adaptor protein 1 |
| 0.03980 | 1.441 | 22.6 | 32.6 | RNF130 | ring finger protein 130 |
| 0.03981 | 1.526 | 7.3 | 11.1 | PLK3 | polo-like kinase 3 |
| 0.03988 | 1.256 | 6.6 | 8.3 | SIL1 | SIL1 nucleotide exchange factor |
| 0.03991 | 1.430 | 5.5 | 7.8 | EHD3 | EH-domain containing 3 |
| 0.03992 | 0.802 | 21.5 | 17.2 | RSF1 | remodeling and spacing factor 1 |
| 0.03992 | 0.730 | 25.3 | 18.5 | DLGAP4 | discs, large (Drosophila) homolog-associated protein 4 |
| 0.03996 | 0.714 | 13.9 | 9.9 | ARID2 | AT rich interactive domain 2 (ARID, RFX-like) |
| 0.03998 | 2.057 | 9.0 | 18.4 | OAS3 | 2'-5'-oligoadenylate synthetase 3, 100kDa |
| 0.04002 | 1.168 | 7.6 | 8.9 | MIS12 | MIS12 kinetochore complex component |
| 0.04004 | 2.423 | 9.7 | 23.5 | FGFR2 | fibroblast growth factor receptor 2 |
| 0.04005 | 1.215 | 14.5 | 17.6 | VAPA | VAMP (vesicle-associated membrane protein)-associated protein A, 33kDa |
| 0.04008 | 1.618 | 10.7 | 17.3 | NACAD | NAC alpha domain containing |
| 0.04011 | 1.580 | 5.0 | 7.9 | SLC47A2 | solute carrier family 47 (multidrug and toxin extrusion), member 2 |
| 0.04013 | 2.597 | 8.5 | 22.2 | CCL3 | chemokine (C-C motif) ligand 3 |
| 0.04014 | 0.673 | 9.0 | 6.0 | FAM106B | family with sequence similarity 106, member B |
| 0.04015 | 0.833 | 6.4 | 5.3 | LOC102725166 | uncharacterized LOC102725166 |
| 0.04023 | 0.795 | 12.6 | 10.0 | PAPD7 | PAP associated domain containing 7 |
| 0.04030 | 0.780 | 22.6 | 17.6 | RBM8A | RNA binding motif protein 8A |
| 0.04045 | 1.470 | 20.1 | 29.6 | CXCL16 | chemokine (C-X-C motif) ligand 16 |
| 0.04048 | 0.867 | 21.4 | 18.6 | C17orf85 | chromosome 17 open reading frame 85 |
| 0.04049 | 0.800 | 8.4 | 6.7 | ZNF549 | zinc finger protein 549 |
| 0.04050 | 1.490 | 5.2 | 7.8 | LOC101060789 | low affinity immunoglobulin gamma Fc region receptor III-A-like |
| 0.04062 | 0.689 | 14.7 | 10.2 | TOMM40 | translocase of outer mitochondrial membrane 40 homolog (yeast) |
| 0.04064 | 1.261 | 23.8 | 30.0 | GRB2 | growth factor receptor-bound protein 2 |
| 0.04088 | 0.777 | 18.7 | 14.5 | PABPN1 | poly(A) binding protein, nuclear 1 |
| 0.04096 | 1.259 | 14.8 | 18.6 | KIF1C | kinesin family member 1C |
| 0.04103 | 1.255 | 13.6 | 17.0 | C20orf194 | chromosome 20 open reading frame 194 |
| 0.04103 | 1.654 | 12.3 | 20.3 | MXI1 | MAX interactor 1, dimerization protein |
| 0.04104 | 1.360 | 6.7 | 9.1 | SGMS1 | sphingomyelin synthase 1 |
| 0.04105 | 0.763 | 7.6 | 5.8 | ING1 | inhibitor of growth family, member 1 |
| 0.04134 | 0.793 | 8.3 | 6.6 | GPHN | gephyrin |
| 0.04137 | 0.663 | 8.2 | 5.4 | ATAD5 | ATPase family, AAA domain containing 5 |
| 0.04143 | 1.310 | 13.3 | 17.4 | ABHD17A | abhydrolase domain containing 17A |
| 0.04147 | 1.251 | 5.7 | 7.2 | LOC102725330 | uncharacterized LOC102725330 |
| 0.04154 | 0.701 | 38.8 | 27.2 | CDH2 | cadherin 2, type 1, N-cadherin (neuronal) |
| 0.04162 | 0.840 | 26.6 | 22.3 | SSU72 | SSU72 RNA polymerase II CTD phosphatase homolog (S. cerevisiae) |
| 0.04167 | 2.372 | 6.2 | 14.6 | OAS1 | 2'-5'-oligoadenylate synthetase 1, 40/46kDa |
| 0.04173 | 0.754 | 10.9 | 8.2 | SOBP | sine oculis binding protein homolog (Drosophila) |
| 0.04176 | 1.314 | 5.0 | 6.6 | DFNB31 | deafness, autosomal recessive 31 |
| 0.04182 | 0.757 | 13.1 | 9.9 | ACBD6 | acyl-CoA binding domain containing 6 |
| 0.04185 | 0.564 | 9.2 | 5.2 | MIR3125 | microRNA 3125 |
| 0.04186 | 1.280 | 6.7 | 8.6 | PRPF18 | pre-mRNA processing factor 18 |
| 0.04188 | 1.370 | 5.1 | 6.9 | PSD | pleckstrin and Sec7 domain containing |
| 0.04194 | 1.316 | 5.5 | 7.2 | NFATC2 | nuclear factor of activated T-cells, cytoplasmic, calcineurin-dependent 2 |
| 0.04198 | 1.173 | 9.4 | 11.1 | TDP2 | tyrosyl-DNA phosphodiesterase 2 |
| 0.04202 | 1.796 | 5.2 | 9.3 | ASPA | aspartoacylase |
| 0.04205 | 0.797 | 6.4 | 5.1 | ZNF567 | zinc finger protein 567 |
| 0.04208 | 0.599 | 12.1 | 7.2 | COL9A3 | collagen, type IX, alpha 3 |
| 0.04211 | 1.248 | 9.0 | 11.2 | CNPPD1 | cyclin Pas1/PHO80 domain containing 1 |
| 0.04214 | 1.167 | 6.4 | 7.4 | ENGASE | endo-beta-N-acetylglucosaminidase |
| 0.04214 | 0.733 | 10.1 | 7.4 | LOC101930634 | uncharacterized LOC101930634 |
| 0.04220 | 1.730 | 5.9 | 10.2 | KCNMA1 | potassium large conductance calcium-activated channel, subfamily M, alpha member 1 |
| 0.04230 | 1.259 | 20.8 | 26.2 | TRIM8 | tripartite motif containing 8 |
| 0.04232 | 0.757 | 15.9 | 12.1 | MCM3AP-AS1 | MCM3AP antisense RNA 1 |
| 0.04232 | 1.837 | 6.5 | 12.0 | SYNDIG1 | synapse differentiation inducing 1 |
| 0.04240 | 1.938 | 8.7 | 16.9 | NAPB | N-ethylmaleimide-sensitive factor attachment protein, beta |
| 0.04248 | 0.794 | 18.9 | 15.0 | BRD8 | bromodomain containing 8 |
| 0.04256 | 1.568 | 8.7 | 13.6 | AGTPBP1 | ATP/GTP binding protein 1 |
| 0.04257 | 0.728 | 15.2 | 11.1 | RFX7 | regulatory factor X, 7 |
| 0.04261 | 0.664 | 40.6 | 26.9 | SOX9 | SRY (sex determining region Y)-box 9 |
| 0.04271 | 0.675 | 25.4 | 17.1 | SLC16A1-AS1 | SLC16A1 antisense RNA 1 |
| 0.04274 | 0.816 | 6.4 | 5.2 | SRSF12 | serine/arginine-rich splicing factor 12 |
| 0.04290 | 0.694 | 52.7 | 36.5 | SETD5 | SET domain containing 5 |
| 0.04304 | 1.323 | 5.0 | 6.7 | RAPGEF3 | Rap guanine nucleotide exchange factor (GEF) 3 |
| 0.04309 | 1.325 | 5.0 | 6.6 | AC018647.3 | uncharacterized LOC100506725 |
| 0.04310 | 0.796 | 32.9 | 26.2 | WHSC1L1 | Wolf-Hirschhorn syndrome candidate 1-like 1 |
| 0.04317 | 1.163 | 5.0 | 5.8 | IKZF2 | IKAROS family zinc finger 2 (Helios) |
| 0.04321 | 0.750 | 11.8 | 8.8 | NIFK | nucleolar protein interacting with the FHA domain of MKI67 |
| 0.04329 | 1.314 | 6.1 | 8.0 | SLC9A6 | solute carrier family 9, subfamily A (NHE6, cation proton antiporter 6), member 6 |
| 0.04329 | 0.256 | 80.9 | 20.7 | NCAN | neurocan |
| 0.04338 | 0.823 | 18.1 | 14.9 | SAFB2 | scaffold attachment factor B2 |
| 0.04351 | 0.715 | 26.0 | 18.6 | FLJ44342 | uncharacterized LOC645460 |
| 0.04357 | 1.206 | 5.2 | 6.3 | EBP | emopamil binding protein (sterol isomerase) |
| 0.04358 | 1.517 | 107.0 | 162.2 | ALDOA | aldolase A, fructose-bisphosphate |
| 0.04360 | 2.171 | 8.8 | 19.0 | CAPN3 | calpain 3, (p94) |
| 0.04360 | 0.752 | 29.2 | 22.0 | CAMSAP2 | calmodulin regulated spectrin-associated protein family, member 2 |
| 0.04360 | 1.481 | 6.1 | 9.1 | HPRT1 | hypoxanthine phosphoribosyltransferase 1 |
| 0.04379 | 1.827 | 14.3 | 26.1 | PXK | PX domain containing serine/threonine kinase |
| 0.04381 | 0.866 | 5.9 | 5.1 | RPAP1 | RNA polymerase II associated protein 1 |
| 0.04382 | 1.298 | 15.2 | 19.7 | SELT | selenoprotein T |
| 0.04387 | 1.588 | 65.0 | 103.3 | S100B | S100 calcium binding protein B |
| 0.04391 | 1.304 | 5.8 | 7.6 | TMEM141 | transmembrane protein 141 |
| 0.04403 | 0.818 | 13.6 | 11.1 | KLHL42 | kelch-like family member 42 |
| 0.04406 | 0.704 | 7.6 | 5.4 | FGF14-IT1 | FGF14 intronic transcript 1 (non-protein coding) |
| 0.04407 | 1.564 | 9.6 | 15.0 | NME7 | NME/NM23 family member 7 |
| 0.04420 | 0.754 | 7.0 | 5.3 | ING2 | inhibitor of growth family, member 2 |
| 0.04421 | 1.734 | 18.7 | 32.4 | SLC48A1 | solute carrier family 48 (heme transporter), member 1 |
| 0.04424 | 0.821 | 6.9 | 5.7 | TSPAN11 | tetraspanin 11 |
| 0.04425 | 0.812 | 12.6 | 10.2 | ZNF880 | zinc finger protein 880 |
| 0.04426 | 1.505 | 13.9 | 20.9 | MEF2C | myocyte enhancer factor 2C |
| 0.04432 | 1.466 | 24.9 | 36.4 | SIRPA | signal-regulatory protein alpha |
| 0.04438 | 1.616 | 7.9 | 12.7 | SP110 | SP110 nuclear body protein |
| 0.04443 | 0.840 | 6.2 | 5.2 | RP4-640H8.2 | uncharacterized LOC100287792 |
| 0.04444 | 1.332 | 6.5 | 8.7 | TECPR2 | tectonin beta-propeller repeat containing 2 |
| 0.04445 | 2.918 | 6.7 | 19.7 | ISG15 | ISG15 ubiquitin-like modifier |
| 0.04450 | 0.576 | 15.6 | 9.0 | UHRF1 | ubiquitin-like with PHD and ring finger domains 1 |
| 0.04450 | 1.722 | 5.1 | 8.7 | NKAIN2 | Na+/K+ transporting ATPase interacting 2 |
| 0.04451 | 1.218 | 7.0 | 8.5 | GABPA | GA binding protein transcription factor, alpha subunit 60kDa |
| 0.04459 | 0.749 | 15.2 | 11.4 | GNPAT | glyceronephosphate O-acyltransferase |
| 0.04461 | 1.894 | 39.7 | 75.2 | ANXA1 | annexin A1 |
| 0.04461 | 0.807 | 7.3 | 5.9 | LOC100507373 | uncharacterized LOC100507373 |
| 0.04464 | 0.712 | 7.2 | 5.1 | CENPJ | centromere protein J |
| 0.04474 | 1.460 | 9.1 | 13.3 | COPRS | coordinator of PRMT5, differentiation stimulator |
| 0.04477 | 1.270 | 11.7 | 14.9 | ARL1 | ADP-ribosylation factor-like 1 |
| 0.04488 | 1.644 | 7.5 | 12.4 | LYN | v-yes-1 Yamaguchi sarcoma viral related oncogene homolog |
| 0.04504 | 1.810 | 10.1 | 18.3 | GSN-AS1 | GSN antisense RNA 1 |
| 0.04506 | 0.827 | 27.4 | 22.6 | CDC42BPA | CDC42 binding protein kinase alpha (DMPK-like) |
| 0.04509 | 0.835 | 20.9 | 17.4 | CCAR1 | cell division cycle and apoptosis regulator 1 |
| 0.04510 | 1.689 | 28.7 | 48.5 | SGK1 | serum/glucocorticoid regulated kinase 1 |
| 0.04516 | 0.657 | 8.4 | 5.5 | NAV2-AS5 | NAV2 antisense RNA 5 |
| 0.04531 | 0.815 | 6.1 | 5.0 | LOC101927886 | uncharacterized LOC101927886 |
| 0.04541 | 0.806 | 6.3 | 5.1 | TMEM133 | transmembrane protein 133 |
| 0.04544 | 1.261 | 15.1 | 19.0 | GCC2 | GRIP and coiled-coil domain containing 2 |
| 0.04545 | 0.864 | 23.1 | 19.9 | EIF3J | eukaryotic translation initiation factor 3, subunit J |
| 0.04545 | 1.207 | 8.0 | 9.7 | PDHX | pyruvate dehydrogenase complex, component X |
| 0.04551 | 0.775 | 29.5 | 22.9 | SRGAP2 | SLIT-ROBO Rho GTPase activating protein 2 |
| 0.04567 | 0.799 | 17.0 | 13.6 | AKT2 | v-akt murine thymoma viral oncogene homolog 2 |
| 0.04573 | 0.606 | 11.2 | 6.8 | ARAP3 | ArfGAP with RhoGAP domain, ankyrin repeat and PH domain 3 |
| 0.04577 | 0.886 | 6.2 | 5.5 | SENP1 | SUMO1/sentrin specific peptidase 1 |
| 0.04578 | 1.497 | 8.4 | 12.5 | MYO1E | myosin IE |
| 0.04583 | 0.745 | 12.0 | 9.0 | PROSER1 | proline and serine rich 1 |
| 0.04584 | 1.405 | 16.7 | 23.5 | TMBIM1 | transmembrane BAX inhibitor motif containing 1 |
| 0.04589 | 0.730 | 14.4 | 10.5 | ZNF789 | zinc finger protein 789 |
| 0.04590 | 1.554 | 5.7 | 8.9 | MPEG1 | macrophage expressed 1 |
| 0.04593 | 1.367 | 9.5 | 13.0 | AKNA | AT-hook transcription factor |
| 0.04594 | 1.944 | 5.1 | 9.8 | C10orf90 | chromosome 10 open reading frame 90 |
| 0.04596 | 1.307 | 21.3 | 27.8 | ATP6V0A1 | ATPase, H+ transporting, lysosomal V0 subunit a1 |
| 0.04600 | 4.294 | 6.4 | 27.5 | CNDP1 | carnosine dipeptidase 1 (metallopeptidase M20 family) |
| 0.04607 | 1.484 | 6.0 | 8.9 | CYP27A1 | cytochrome P450, family 27, subfamily A, polypeptide 1 |
| 0.04611 | 0.676 | 40.5 | 27.4 | LHFP | lipoma HMGIC fusion partner |
| 0.04623 | 1.237 | 6.7 | 8.3 | TUBGCP2 | tubulin, gamma complex associated protein 2 |
| 0.04624 | 1.347 | 5.2 | 7.0 | SNPH | syntaphilin |
| 0.04625 | 0.830 | 6.0 | 5.0 | ARHGAP11B | Rho GTPase activating protein 11B |
| 0.04626 | 1.601 | 9.0 | 14.4 | SPI1 | spleen focus forming virus (SFFV) proviral integration oncogene |
| 0.04628 | 0.521 | 30.6 | 15.9 | GRIK3 | glutamate receptor, ionotropic, kainate 3 |
| 0.04631 | 1.613 | 34.5 | 55.7 | SLCO2B1 | solute carrier organic anion transporter family, member 2B1 |
| 0.04632 | 1.289 | 5.7 | 7.3 | WDR37 | WD repeat domain 37 |
| 0.04637 | 1.175 | 5.0 | 5.9 | LINC00601 | long intergenic non-protein coding RNA 601 |
| 0.04642 | 1.167 | 5.1 | 6.0 | LZTFL1 | leucine zipper transcription factor-like 1 |
| 0.04644 | 1.270 | 9.9 | 12.6 | NTPCR | nucleoside-triphosphatase, cancer-related |
| 0.04648 | 1.519 | 8.1 | 12.4 | FRMD4B | FERM domain containing 4B |
| 0.04658 | 1.233 | 10.6 | 13.0 | CLCN7 | chloride channel, voltage-sensitive 7 |
| 0.04661 | 0.518 | 10.9 | 5.7 | MYT1 | myelin transcription factor 1 |
| 0.04666 | 3.051 | 6.6 | 20.3 | VSNL1 | visinin-like 1 |
| 0.04666 | 0.744 | 7.8 | 5.8 | NUP35 | nucleoporin 35kDa |
| 0.04674 | 0.833 | 9.1 | 7.6 | UTP14C | UTP14, U3 small nucleolar ribonucleoprotein, homolog C (yeast) |
| 0.04683 | 1.223 | 6.1 | 7.5 | SLC17A5 | solute carrier family 17 (acidic sugar transporter), member 5 |
| 0.04685 | 0.713 | 18.8 | 13.4 | DDTL | D-dopachrome tautomerase-like |
| 0.04689 | 1.305 | 118.3 | 154.3 | DPYSL2 | dihydropyrimidinase-like 2 |
| 0.04692 | 1.489 | 18.5 | 27.6 | MFSD1 | major facilitator superfamily domain containing 1 |
| 0.04692 | 0.850 | 6.0 | 5.1 | ZNF607 | zinc finger protein 607 |
| 0.04697 | 0.851 | 20.6 | 17.5 | GIT2 | G protein-coupled receptor kinase interacting ArfGAP 2 |
| 0.04698 | 0.692 | 26.5 | 18.4 | PPP1R14B | protein phosphatase 1, regulatory (inhibitor) subunit 14B |
| 0.04698 | 0.773 | 18.5 | 14.3 | C1orf174 | chromosome 1 open reading frame 174 |
| 0.04699 | 1.179 | 5.1 | 6.1 | CNNM2 | cyclin M2 |
| 0.04703 | 1.483 | 10.6 | 15.7 | PVRL1 | poliovirus receptor-related 1 (herpesvirus entry mediator C) |
| 0.04703 | 1.453 | 10.6 | 15.5 | MFSD6 | major facilitator superfamily domain containing 6 |
| 0.04704 | 0.743 | 7.7 | 5.7 | DGKH | diacylglycerol kinase, eta |
| 0.04705 | 1.334 | 20.4 | 27.2 | RAP1A | RAP1A, member of RAS oncogene family |
| 0.04714 | 0.751 | 23.6 | 17.8 | SNX22 | sorting nexin 22 |
| 0.04720 | 0.591 | 54.7 | 32.3 | RNU6-15P | RNA, U6 small nuclear 15, pseudogene |
| 0.04721 | 2.040 | 5.0 | 10.2 | NECAB1 | N-terminal EF-hand calcium binding protein 1 |
| 0.04731 | 1.688 | 9.9 | 16.8 | EVI2A | ecotropic viral integration site 2A |
| 0.04736 | 0.625 | 9.1 | 5.7 | RFC3 | replication factor C (activator 1) 3, 38kDa |
| 0.04739 | 0.774 | 7.4 | 5.7 | SERHL2 | serine hydrolase-like 2 |
| 0.04746 | 1.240 | 25.7 | 31.8 | BACE1 | beta-site APP-cleaving enzyme 1 |
| 0.04747 | 1.543 | 10.8 | 16.7 | SNORA71B | small nucleolar RNA, H/ACA box 71B |
| 0.04750 | 1.467 | 11.7 | 17.1 | SNURF | SNRPN upstream reading frame |
| 0.04754 | 1.543 | 5.0 | 7.7 | LACC1 | laccase (multicopper oxidoreductase) domain containing 1 |
| 0.04757 | 0.794 | 7.7 | 6.1 | C19orf44 | chromosome 19 open reading frame 44 |
| 0.04764 | 0.818 | 9.1 | 7.5 | OGFOD3 | 2-oxoglutarate and iron-dependent oxygenase domain containing 3 |
| 0.04766 | 1.327 | 14.2 | 18.8 | ERAP1 | endoplasmic reticulum aminopeptidase 1 |
| 0.04780 | 1.168 | 5.2 | 6.1 | LOC339803 | uncharacterized LOC339803 |
| 0.04782 | 0.742 | 6.9 | 5.1 | C16orf87 | chromosome 16 open reading frame 87 |
| 0.04782 | 1.299 | 10.7 | 14.0 | CCNY | cyclin Y |
| 0.04784 | 0.782 | 7.6 | 6.0 | ZNF444 | zinc finger protein 444 |
| 0.04787 | 1.329 | 9.8 | 13.0 | RAB18 | RAB18, member RAS oncogene family |
| 0.04787 | 0.547 | 393.6 | 215.3 | PTPRZ1 | protein tyrosine phosphatase, receptor-type, Z polypeptide 1 |
| 0.04793 | 0.750 | 18.4 | 13.8 | SMARCAD1 | SWI/SNF-related, matrix-associated actin-dependent regulator of chromatin, subfamily a, containing DEAD/H box 1 |
| 0.04794 | 0.802 | 16.3 | 13.1 | MSH6 | mutS homolog 6 |
| 0.04794 | 0.760 | 51.0 | 38.8 | EIF4G3 | eukaryotic translation initiation factor 4 gamma, 3 |
| 0.04801 | 0.777 | 10.6 | 8.3 | BCL9L | B-cell CLL/lymphoma 9-like |
| 0.04804 | 1.951 | 50.5 | 98.5 | NTRK2 | neurotrophic tyrosine kinase, receptor, type 2 |
| 0.04811 | 1.869 | 5.0 | 9.3 | CREG2 | cellular repressor of E1A-stimulated genes 2 |
| 0.04819 | 2.052 | 14.0 | 28.6 | DAB2 | Dab, mitogen-responsive phosphoprotein, homolog 2 (Drosophila) |
| 0.04823 | 1.616 | 15.0 | 24.2 | ARHGAP23 | Rho GTPase activating protein 23 |
| 0.04825 | 1.254 | 6.1 | 7.7 | HAGH | hydroxyacylglutathione hydrolase |
| 0.04834 | 0.692 | 8.1 | 5.6 | LOC100505767 | putative speedy protein-like protein LOC442572-like |
| 0.04835 | 1.363 | 10.3 | 14.0 | SCHIP1 | schwannomin interacting protein 1 |
| 0.04836 | 0.744 | 24.5 | 18.2 | RPAIN | RPA interacting protein |
| 0.04841 | 0.772 | 8.8 | 6.8 | LOC100133091 | uncharacterized LOC100133091 |
| 0.04842 | 0.724 | 25.8 | 18.7 | AC012309.5 | uncharacterized LOC101927667 |
| 0.04844 | 1.323 | 6.3 | 8.3 | APIP | APAF1 interacting protein |
| 0.04847 | 1.296 | 5.8 | 7.5 | ALDH3B1 | aldehyde dehydrogenase 3 family, member B1 |
| 0.04851 | 0.815 | 6.2 | 5.0 | SNORD62B | small nucleolar RNA, C/D box 62B |
| 0.04852 | 0.855 | 15.5 | 13.3 | UBTF | upstream binding transcription factor, RNA polymerase I |
| 0.04854 | 1.562 | 5.0 | 7.8 | LDB3 | LIM domain binding 3 |
| 0.04860 | 0.635 | 9.5 | 6.0 | RNF165 | ring finger protein 165 |
| 0.04866 | 1.349 | 5.8 | 7.9 | CRY2 | cryptochrome circadian clock 2 |
| 0.04871 | 1.355 | 5.7 | 7.7 | GAREM | GRB2 associated, regulator of MAPK1 |
| 0.04874 | 0.703 | 13.2 | 9.3 | ZNF316 | zinc finger protein 316 |
| 0.04890 | 1.455 | 6.8 | 9.9 | CLSTN3 | calsyntenin 3 |
| 0.04898 | 1.422 | 16.2 | 23.0 | ALDH3A2 | aldehyde dehydrogenase 3 family, member A2 |
| 0.04902 | 0.657 | 8.3 | 5.4 | XYLT1 | xylosyltransferase I |
| 0.04903 | 0.731 | 21.0 | 15.3 | CPSF6 | cleavage and polyadenylation specific factor 6, 68kDa |
| 0.04904 | 1.959 | 18.1 | 35.4 | LOC102724761 | uncharacterized LOC102724761 |
| 0.04906 | 1.250 | 18.0 | 22.5 | ALKBH5 | alkB, alkylation repair homolog 5 (E. coli) |
| 0.04911 | 0.691 | 13.4 | 9.3 | STRA13 | stimulated by retinoic acid 13 |
| 0.04911 | 0.622 | 9.4 | 5.8 | HELLS | helicase, lymphoid-specific |
| 0.04917 | 0.764 | 16.7 | 12.8 | MPHOSPH9 | M-phase phosphoprotein 9 |
| 0.04918 | 0.662 | 39.2 | 25.9 | EPN2 | epsin 2 |
| 0.04919 | 0.594 | 58.8 | 34.9 | LINC00461 | long intergenic non-protein coding RNA 461 |
| 0.04925 | 0.641 | 10.0 | 6.4 | TLE2 | transducin-like enhancer of split 2 |
| 0.04926 | 1.280 | 5.1 | 6.5 | C21orf91 | chromosome 21 open reading frame 91 |
| 0.04930 | 1.238 | 15.5 | 19.2 | YPEL5 | yippee-like 5 (Drosophila) |
| 0.04934 | 1.180 | 11.7 | 13.8 | KIAA0196 | KIAA0196 |
| 0.04936 | 1.228 | 5.2 | 6.4 | TTC18 | tetratricopeptide repeat domain 18 |
| 0.04938 | 0.798 | 26.0 | 20.7 | PDCD5 | programmed cell death 5 |
| 0.04940 | 1.308 | 12.1 | 15.8 | RABAC1 | Rab acceptor 1 (prenylated) |
| 0.04943 | 0.842 | 6.3 | 5.3 | LOC100379224 | uncharacterized LOC100379224 |
| 0.04944 | 0.832 | 13.3 | 11.0 | ZNF384 | zinc finger protein 384 |
| 0.04951 | 1.967 | 7.9 | 15.6 | NEK7 | NIMA-related kinase 7 |
| 0.04952 | 1.571 | 5.0 | 7.9 | FA2H | fatty acid 2-hydroxylase |
| 0.04953 | 1.885 | 5.0 | 9.4 | GDA | guanine deaminase |
| 0.04954 | 0.746 | 7.7 | 5.7 | PI4KAP1 | phosphatidylinositol 4-kinase, catalytic, alpha pseudogene 1 |
| 0.04956 | 0.757 | 28.7 | 21.7 | SRRT | serrate RNA effector molecule homolog (Arabidopsis) |
| 0.04958 | 0.524 | 13.0 | 6.8 | THSD7A | thrombospondin, type I, domain containing 7A |
| 0.04963 | 0.794 | 18.6 | 14.7 | CDK13 | cyclin-dependent kinase 13 |
| 0.04966 | 0.800 | 10.6 | 8.4 | RHNO1 | RAD9-HUS1-RAD1 interacting nuclear orphan 1 |
| 0.04970 | 0.760 | 9.1 | 6.9 | ZNF782 | zinc finger protein 782 |
| 0.04975 | 0.727 | 7.2 | 5.2 | ZFP69B | ZFP69 zinc finger protein B |
| 0.04992 | 1.878 | 5.2 | 9.9 | SH3GL3 | SH3-domain GRB2-like 3 |
| 0.04996 | 1.305 | 13.0 | 16.9 | ARAP1 | ArfGAP with RhoGAP domain, ankyrin repeat and PH domain 1 |
| 0.04999 | 0.817 | 6.1 | 5.0 | LINC00662 | long intergenic non-protein coding RNA 662 |
|  |  |  |  |  |  |
